# Changes in Retinal Structure and Ultrastructure in the Aged Mice Correlate With Differences in the Expression of Selected Retinal miRNAs

**DOI:** 10.3389/fphar.2020.593514

**Published:** 2021-01-13

**Authors:** Anca Hermenean, Maria Consiglia Trotta, Sami Gharbia, Andrei Gelu Hermenean, Victor Eduard Peteu, Cornel Balta, Coralia Cotoraci, Carlo Gesualdo, Settimio Rossi, Mihaela Gherghiceanu, Michele D’Amico

**Affiliations:** ^1^ “Aurel Ardelean” Institute of Life Sciences, Vasile Goldis Western University of Arad, Arad, Romania; ^2^ Department of Biochemistry and Molecular Biology, University of Bucharest, Bucharest, Romania; ^3^ Section of Pharmacology, Department of Experimental Medicine, University of Campania “Luigi Vanvitelli”, Naples, Italy; ^4^ Carol Davila University of Medicine and Pharmacy, Bucharest, Romania; ^5^ Victor Babes National Institute of Pathology, Bucharest, Romania; ^6^ Faculty of Medicine, Vasile Goldis Western University of Arad, Arad, Romania; ^7^ Eye Clinic, Multidisciplinary Department of Medical, Surgical and Dental Sciences, University of Campania “Luigi Vanvitelli”, Naples, Italy

**Keywords:** aging, retina, gender, histology, electron micoscopy, miRNAs

## Abstract

Age and gender are two important factors that may influence the function and structure of the retina and its susceptibility to retinal diseases. The aim of this study was to delineate the influence that biological sex and age exert on the retinal structural and ultrastructural changes in mice and to identify the age-related miRNA dysregulation profiles in the retina by gender. Experiments were undertaken on male and female Balb/c aged 24 months (approximately 75–85 years in humans) compared to the control (3 months). The retinas were analyzed by histology, transmission electron microscopy, and age-related miRNA expression profile analysis. Retinas of both sexes showed a steady decline in retinal thickness as follows: photoreceptor (PS) and outer layers (*p* < 0.01 for the aged male vs. control; *p* < 0.05 for the aged female vs. control); the inner retinal layers were significantly affected by the aging process in the males (*p* < 0.01) but not in the aged females. Electron microscopy revealed more abnormalities which involve the retinal pigment epithelium (RPE) and Bruch’s membrane, outer and inner layers, vascular changes, deposits of amorphous materials, and accumulation of lipids or lipofuscins. Age-related miRNAs, miR-27a-3p (*p* < 0.01), miR-27b-3p (*p* < 0.05), and miR-20a-5p (*p* < 0.05) were significantly up-regulated in aged male mice compared to the controls, whereas miR-20b-5p was significantly down-regulated in aged male (*p* < 0.05) and female mice (*p* < 0.05) compared to the respective controls. miR-27a-3p (5.00 fold; *p* < 0.01) and miR-27b (7.58 fold; *p* < 0.01) were significantly up-regulated in aged male mice vs. aged female mice, whereas miR-20b-5p (−2.10 fold; *p* < 0.05) was significantly down-regulated in aged male mice vs. aged female mice. Interestingly, miR-27a-3p, miR-27b-3p, miR-20a-5p, and miR-20b-5p expressions significantly correlated with the thickness of the retinal PS layer (*p* < 0.01), retinal outer layers (*p* < 0.01), and Bruch’s membrane (*p* < 0.01). Our results showed that biological sex can influence the structure and function of the retina upon aging, suggesting that this difference may be underlined by the dysregulation of age-related mi-RNAs.

## Introduction

The structural and functional physiological evolution of the retina with age can be influenced by biological sex and susceptibility to retinal diseases (Wagner et al., 2008; Freund et al., 2011). There are anatomical sex differences, visual performances, and divergent molecular profiles (Du et al., 2017) by gender upon aging, which may contribute to different susceptibilities to disease and trace different evolutions for retinal pathologies (Wagner et al., 2008; Ozawa et al., 2015; Schmidl et al., 2015). Numerous eye anatomical changes occur with age, including cell loss (e.g., corneal and trabecular endothelium and retinal pigment epithelium [RPE]) and degenerative processes (e.g., vitreous liquefaction and drusen) (Grossniklaus et al., 2013), that may be influenced by gender differences. Clinical results have reported that women have thinner retinas than men, without any differences in the foveal pit morphology (Wagner-Schuman et al., 2011), whereas the amplitudes of scotopic and photopic electroretinograms (ERGs) of female subjects have been reported to be on average 29% larger than those of males (Brûlé et al., 2007). Morphological studies have shown gender-related structural differences in thickness of the macular retinal layers by spectral-domain optical coherence tomography (SD-OCT) (Adhi et al., 2012; Hashamani et al., 2018). Additionally, the outer nuclear layers and the inner nuclear layer (INL) have been determined to be thicker in men, whereas the nerve fiber layer (NFL) is thicker in women (Ooto et al., 2011).

The anatomical and physiological differences of the retina related to gender are reflected in the associated pathologies (Zetterberg, 2016; Nuzzi et al., 2018). Aging is the first risk factor that leads to glaucoma or age-related macular degeneration (AMD). Some studies have shown a higher incidence and severity of late-stage AMD in women (AREDS Research Group, 2000; Chakravarthy et al., 2010), whereas others have not pointed to a gender difference (Buch et al., 2005; Laitinen et al., 2010). One of the main risk factors for developing AMD is related to macular pigment deficiency, which is more pronounced in women (Beatty et al., 2000; Meyers et al., 2013). Cataracts are more prevalent among women aged 65 and 74 years (24–27%) than men of the same age (14–20%) (Klein et al., 1994; Freeman et al., 2001; Younan et al., 2002). Estrogen exerts a protective effect for women due to its antioxidant properties (Beebe et al., 2010); however, after menopause, the age-related hormonal decline increases the risk of cataracts with age (Lai et al., 2013; van den Beld et al., 2018). In contrast, estrogen levels converted from aromatase to testosterone in men do not appear to be related to age, providing greater protection against cataracts (Zettemberg and Celojevic, 2015). Other results suggested that females may have some protection or resistance to neurodegenerative changes prior to retinopathy in type 2 diabetes (Ozawa et al., 2012). One prediction is that females with type 2 diabetes have better vascular perfusion than man due to estrogen because of the increased production of nitric oxide and nitric oxide synthase (NOS) genes (Mendelsohn and Karas, 2005), attenuating retinal ischemia–reperfusion (Nonaka et al., 2000). However, in an Early Treatment Diabetic Retinopathy Study (ETDRS), the female sex was reported to be a risk factor for severe visual impairment or the need to perform a vitrectomy (Trautner et al., 1997; Davis et al., 1998). Upon aging, gender-related retinal changes may be governed by sexual hormones, such as estrogen. Electroretinogram (ERG) and histology investigations on age-related retinal changes have shown the role of biological sex and age in retinal function (Chaychi et al., 2015).

MicroRNAs (miRNAs), a class of short noncoding RNAs, have been identified that are up- or down-regulated during mammalian aging (Smith-Vikos and Slack, 2012), but no study has particularly referred to retinal aging. A few studies on microRNAs have reported on age-related macular degeneration (AMD), which is the most frequent pathology associated with aging. Recently, four studies analyzed the circulating miRNA expression profiles of AMD patients (Ertekin et al., 2014; Grassmann et al., 2014; Szemraj et al., 2015; Menard et al., 2016), but the results did not completely overlap possibly because of the different inclusion criteria or different applied therapeutic protocols. Recent findings showed miRNA dysregulation in AMD of ocular tissues, which demonstrated some similarities with human AMD findings, including miR-146a, miR-17, miR-125b, and miR-155 (Berber et al., 2017).

Limited results are available regarding the ultrastructural retinal changes upon human aging and in age-related retinal diseases (Nag and Wadhwa, 2012) or in preclinical studies, as in a senescence-accelerated mouse model (Majji et al., 2000), and no results highlighting electron microscopy differences by gender were found. To date, no one has investigated whether the dysregulation of sex-differentiated miRNAs could be correlated with retinal changes during aging. The aim of this study was to delineate the influence that biological sex and age have on the retinal structural and ultrastructural changes in mice and to identify the age-related miRNA dysregulation profiles in the retina by gender.

## Materials and Methods

### Animals and Experimental Design

All experimental procedures were conducted in compliance with the European and national regulations for the care and use of animals for scientific purposes. Ethics approval was obtained from the Ethics Committee for research of the Vasile Goldis Western University of Arad (approval no. 135, January 03, 2019).

Experiments were performed on male and female Balb/c that were 3 months (male/female control groups) and 24 months (male/female aged groups) of age, respectively (*n* = 10/each group/sex). The life span of laboratory mice ranges from 2 to 2.5 years; thus, 24 months approximate 75–85 years of age for a human (Dutta and Sengupta, 2016). While control females were under physiological and regular estrus cycle, 24-month-old females were under naturally occurring physiological decline for estrus without any manipulation, according to previous evidence showing a progressive decrease in estrogens in Balb-c females after 15 months of age (Nagasawa et al., 1978). Animals were housed in a 22 °C environment in IVC well-ventilated cages with ad libitum access to normal rat chow and water. Lighting (39 ± 7 lux) was regulated on a 12-h light/dark cycle. Particularly, although room light between 130 and 325 lux has been recommended for animals susceptible to phototoxic retinopathy by the National Research Council (US) Committee for the Update of the Guide for the Care and Use of Laboratory Animals (National Research Council, 2011), and a lower illuminance level (39 ± 7 lux) was used in order to minimize the negative effects of standard vivarium lighting on the aged retina (Bell et al., 2015). All the procedures were conducted under ketamine and xylazine anesthesia.

Each mouse was perfused via the left ventricle with 100 ml of 0.1 M ice-cold phosphate-buffered saline (PBS). To increase the efficiency of perfusion, heparin (5000 IU/ml, a final concentration of 0.1% *v*/*v*) was added to PBS (Bozycki et al., 2018). This served for the collection of one eye for biochemical assays. In the next step, animals’ perfusion was continued with an additional 100 ml of freshly prepared 4% paraformaldehyde (PFA) in PBS for the collection of the remaining eye and investigations as detailed below.

### Histology/Light Microscopy

The eye specimens were fixed in 4% paraformaldehyde and were embedded in paraffin and sectioned at 5 μm, and the sections were stained with hematoxylin and eosin (H&E). The light microscopy images were acquired using an Olympus BX43 microscope, with an XC30 CCD and cellSens Dimension Imaging Software (v 1.10, Olympus, Germany).

### Electron Microscopy

The eye samples were prefixed in 2.7% glutaraldehyde solution (Sigma-Aldrich, St Louis, Missouri) in 0.1 M phosphate buffer, then washed in 0.15 M phosphate buffer (pH 7.2), and postfixed in 2% osmic acid solution (Sigma-Aldrich, St Louis, Missouri) in 0.15 M phosphate buffer. Dehydration was performed in acetone, followed by embedding in the epoxy resin (Epon 812). Thin sections of 70 nm thickness were cut on a Leica EM UC7 ultramicrotome (Leica Microsystems GmbH, Wetzlar, Germany). The double staining of thin sections on grids was performed with solutions of uranyl acetate and lead citrate. The sections were imaged under a TEM (Morgagni268, FEI, Eindhoven, Netherlands) at 80 kV. Data acquisition was performed with a MegaView III CCD using iTEM SIS software (Olympus Soft Imaging Software, Munster, Germany).

### Morphometry

For each mouse, the thickness of the retinal layers (i.e., retinal pigment epithelium [RPE], photoreceptor cells [PS], outer nuclear layer [ONL], outer plexiform layer [OPL], inner nuclear layer [INL], inner plexiform layer [IPL], and retinal ganglion cell layer [RGL]) were measured on ten different histological sections taken from the superior and inferior retinas (central and equatorial) every 300 µm from the optic nerve head (ONH) using CellSens Dimension Imaging Software (v 1.10, Olympus, Germany).

The morphometry on the electron microscopy images was performed for the equatorial retina. The thickness of Bruch’s membrane was measured at three different points on the images (*n* = 50 for each group) acquired at the same magnification (7,100×) using iTEM SIS software (Olympus Soft Imaging Software, Munster, Germany) and exported in Excel format for statistical analysis.

### Isolation and Expression Analysis of Retinal microRNAs

After retina dissection following the method described by Rossi et al. (2016), total RNA, including microRNAs, was isolated from aged mouse retina (*N* = 10 per group) by using the MiRNeasy Mini Kit (Qiagen, Italy), according to the manufacturer’s instructions. An appropriate volume of QIAzol Lysis Reagent (Qiagen, Italy) was used for tissue homogenization, and the miRNA isolation efficiency was monitored by adding Syn-cel-miR-39 miScript miRNA Mimic 5 nM (Qiagen, Italy) to each sample before RNA purification. RNA quality and concentration were determined by a NanoDrop 2000c spectrophotometer (Thermo Scientific, Italy). Mature miRNAs were converted into cDNA by reverse transcription performed by using the MiScript II Reverse Transcription Kit (Qiagen, Italy) and a Gene Amp PCR System 9,700 (Applied Biosystems, United States). The levels of eight miRNAs (mmu-miR-20a-5p, mmu-miR-20a-3p, mmu-miR-20b-5p, mmu-miR-106a-5p, mmu-miR-27a-3p, mmu-miR-27b-3p, mmu-miR-206-3p, and mmu-miR-381-3p), which were previously shown to be time-dependently dysregulated in the diabetic retina (Platania et al., 2019), were analyzed by real-time PCR (qPCR) with a CFX96 Real-Time System C1000 Touch Thermal Cycler (BioRad Laboratories, Inc.). The qPCR triplicate measurement was carried out by using miScript primer assays (MS00001309, MS00001869, MS00001316, MS00011039, MS00001315, MS00001385, MS00001869, and MS00032802; Qiagen, Italy), SYBR Green PCR Master Mix (Qiagen, Italy), and Ce_miR-39-5p as an external control (MS00080247, Qiagen, Italy) for normalization of miRNA expression. CFX Manager™ Software (BioRad Laboratories, Inc.) was used to evaluate the cycle threshold (Ct) values to calculate the ΔCt for each miRNA as ΔCt = Ct miRNA–Ct Ce_miR-39-5p and then miRNA expression as 2^^−ΔCt^. For each miRNA profiled, the fold change expression across two experimental groups was calculated as 2^^−ΔΔCt^ (equal to 2^^−ΔCt^ of group 2/2^^−ΔCt^ of group 1) and then expressed as the fold regulation. In particular, the fold up-regulation was equal to the fold change value, whereas the fold down-regulation was calculated as the negative inverse of the fold change value (Platania et al., 2019).

### Statistical Analysis

Values are expressed as means ± SD of *n* = 5 mice per group for histological and electron microscopy evaluation, while miRNA results were expressed as means ± SD of *n* = 10 mice per group. Statistical significance was assessed by one-way ANOVA, followed by Tukey’s multiple comparisons test. Pearson correlation analysis was used to evaluate the strength of the association between pairs of variables. Statistical analysis was carried out with GraphPad Prism v.6 (GraphPad Software, La Jolla, CA, United States). Differences were considered statistically significant for *p* values <0.05.

## Results

### Age-Related Structural Differences in Both Male and Female Mice

**FIGURE 1:**
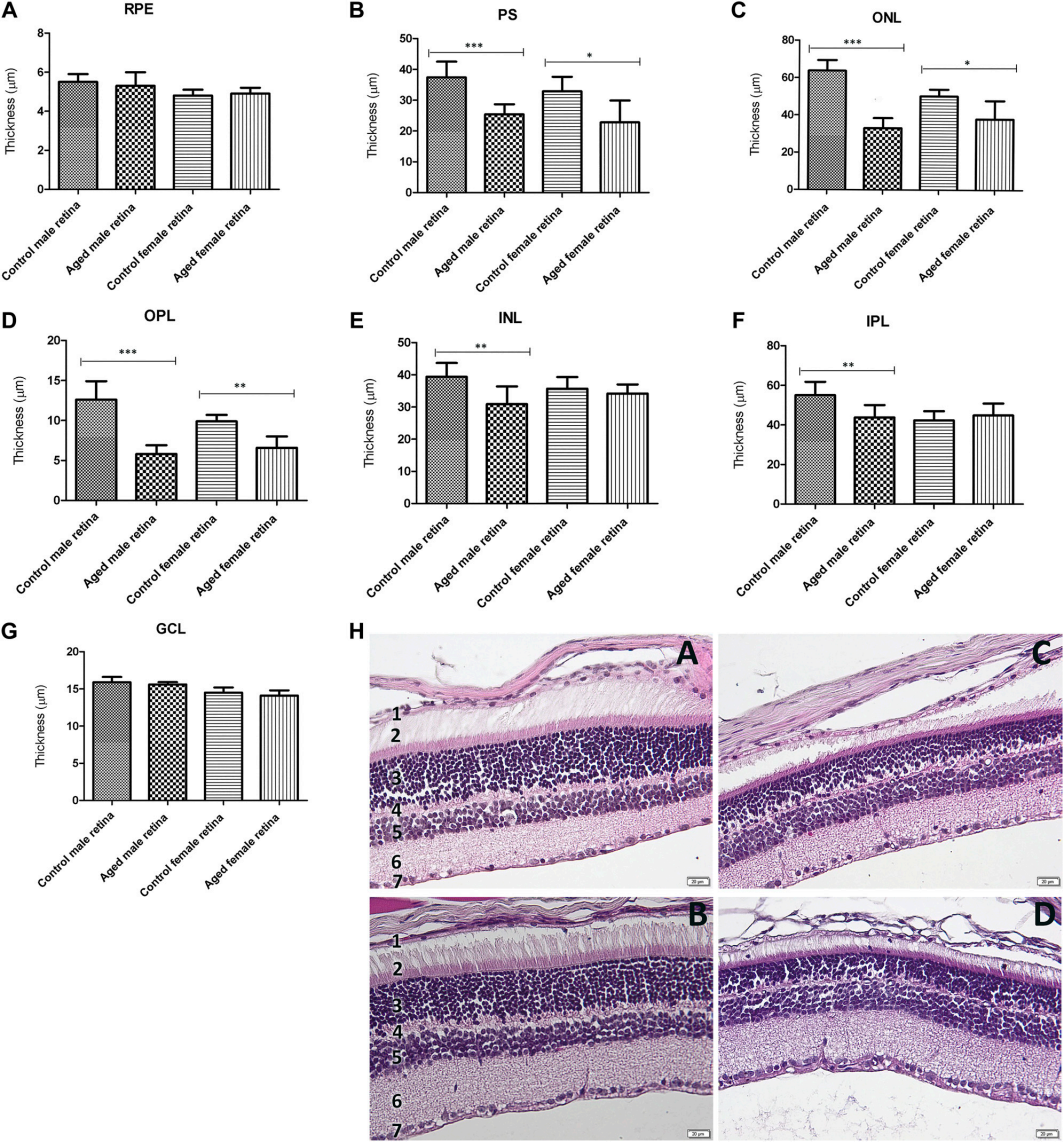
Retinal layer thickness of the aged retinas by gender. **(A)**. The retinal pigment epithelium (RPE) thickness was not significantly modified between aged retinas and controls; **(B)**. photoreceptor outer and inner segment layers (PS) were significantly thinner in both aged males (***p < 0.001 vs. controls) and females (*p < 0.05 vs. controls) compared to their controls, as well as the outer nuclear layer (ONL) **(C)** (aged males ***p < 0.001 vs. controls; aged females *p < 0.05 vs. controls), and the outer plexiform layer (OPL) **(D)** (aged males ***p < 0.001 vs. controls; aged females **p < 0.01 vs. controls). The inner nuclear layer (INL) **(E)** and inner plexiform layer (IPL) **(F)** were both significantly thinner in aged males than the controls (both **p < 0.01 vs. controls), while they did not show any significant differences between aged and control female retina; the retinal ganglion cell layer (RGL) thickness **(G)** was not modified between aged retinas and controls; (h.) histological aspect of the retinal layers of the control male **(A)**, control female **(B)**, aged male **(C)**, and aged female **(D)** showing the reduction in thickness with age for both gender; the legend of the retinal layers: 1. RPE, 2. PS, 3. ONL, 4. OPL, 5. INL, 6. IPL, 7. RGL; thickness (µm) of retinal layers was reported as mean ± SD of *n* = 10 histological observations for each individual/group. Statistical significance was assessed by using the one-way ANOVA, followed by Tukey’s multiple comparisons test.

The aging-induced changes in the entire retinal structure were analyzed. Representative retinal cross sections obtained from male and female mice are shown in Figure 1. As shown in Figures 1A–G, all the retinal layers reduced in thickness with age for both sexes. The photoreceptor outer and inner segment layer (PS), outer nuclear layer (ONL), and outer plexiform layer (OPL) thicknesses in the aged retinas were significantly thinner than those in the control (*p* < 0.01 aged male vs. control; *p* < 0.05 aged female vs. control). The thickness of the INL was reduced 1.27-fold for the males and 1.04 times for the females, whereas the IPL decreased 1.25-fold for males and 1.05-fold for females. The retinal pigment epithelium (RPE) and retinal ganglion cell layer (GGL) reduced approximately onefold in older mice compared to young ones and were similar for both sexes.

### Male and Female Ultrastructural Differences in the Aged Mouse Retina

**FIGURE 2:**
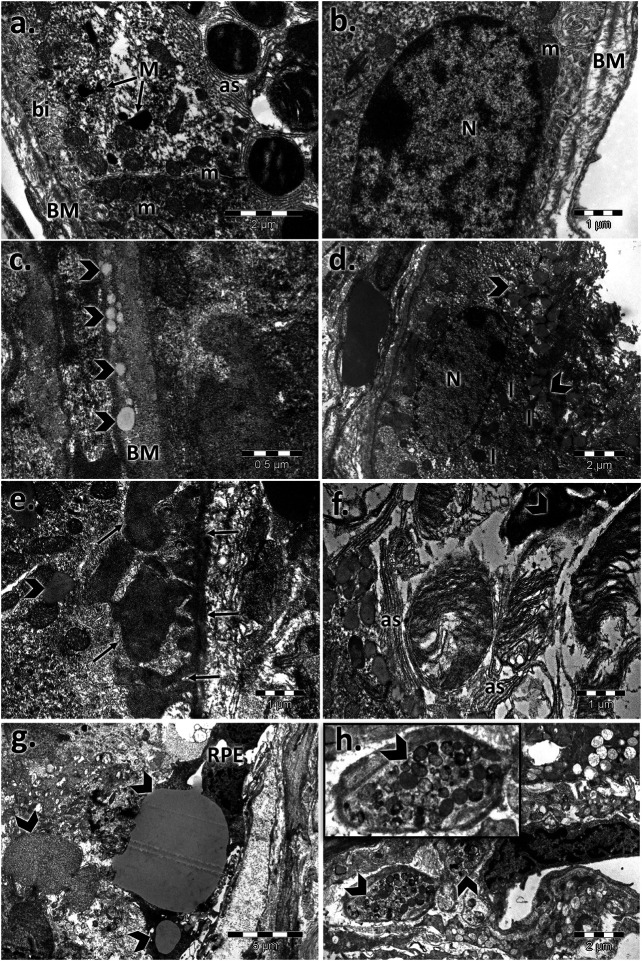
Electron micrographs showing the ultrastructural features of the retinal pigment epithelium (RPE) of the aged male retina. **(A,B)** RPE of the male control, showing the normal aspect of the nucleus (N), mitochondria (m), melanin granules (M), Bruch’s membrane (BM), marked basal infolding (bi), and wide apical sheaths (as) surrounding the photoreceptor outer segments; **(C–H)** RPE of the aged male, showing changes in the RPE ultrastructure, highlighted by thickened Bruch’s membrane (BM) enriched in lipids (arrowhead) **(C)**; lipid drops accumulated in the cytoplasm and crowded in the apical sheath area (arrowhead), lipofuscin (l) **(D)**; localized thickening of Bruch’s membrane on the RPE side and fingerlike extensions of the amorphous material (arrows) **(E)**; atrophic apical sheaths (as) **(F)**; large lipid drops (arrowhead) and atrophied retinal pigment epithelial layer (RPE) **(G)**; clusters of melanosomes and lipofuscin (arrowhead) **(H)**. Figures are representative of *n* = 10 electron micrographs for each individual/group.

**FIGURE 3:**
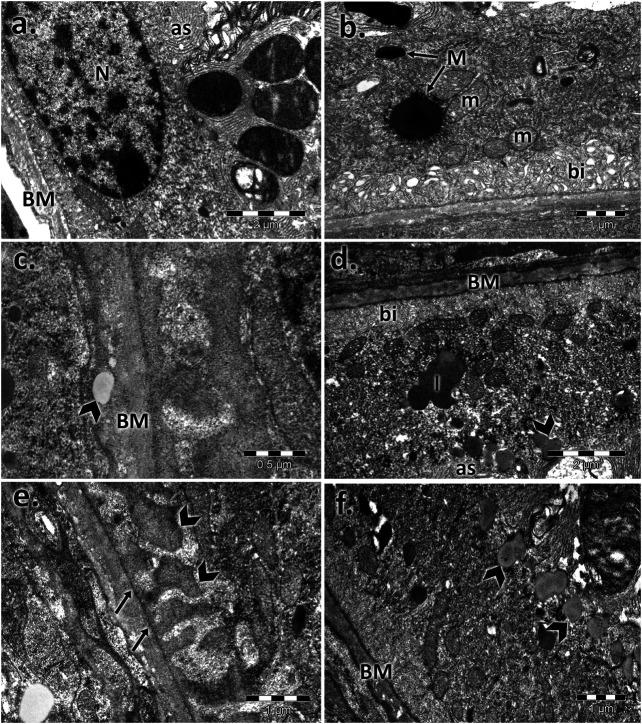
Electron micrographs showing the ultrastructural features of the retinal pigment epithelium (RPE) of the aged female retina. **(A)**–**(B)** RPE of the female control, showing the normal aspect of the nucleus (N), mitochondria (m), melanin granules (M), Bruch’s membrane (BM), marked basal infolding (bi), and wide apical sheaths surrounding the photoreceptor outer segments; **(C)**–**(F)** RPE of the aged female, showing ultrastructural changes of the RPE cells highlighted by the thickened basal membrane (BM), lipid drops into the membrane (arrowhead) **(C)**; lipofuscin granules (l), and lipids (arrowheads) clustered in the apical sheath area (as) **(D)**; localized thickening of Bruch’s membrane on the RPE side and fingerlike extensions of the amorphous material (arrows) **(E)**; lipids (arrowheads) clustered in the apical sheaths area **(F)**. Figures are representative of *n* = 10 electron micrographs for each individual/group.

The control retinal pigment epithelial cells appeared with a normal aspect, separated from the choroid by Bruch’s membrane (BM), showing cytoplasmic melanin granules, mitochondria, and a regular nucleus (Figures 2, 3). The basolateral infoldings of the plasma membrane, the apical villous-like processes, and sheath plaques of the photoreceptor outer segments had a normal aspect. With age, Bruch’s membrane showed significantly increased thickness in both of aged male/female retinas compared to the control (Table 1), and uploading with lipid drops was observed (Figures 2, 3). In particular, we observed localized areas of increased thickness in Bruch’s membrane and abnormal deposits of amorphous material in the sub-RPE space (side of Bruch’s membrane), which was similar to the basal laminar deposits of human AMD. In those areas, we noticed fingerlike extensions of the amorphous material that extended from the basal membrane toward the RPE cytoplasm. The basolateral infolding and apical sheaths were reduced or apparently disorganized in the retinal periphery, mainly for the aged male retina. The cells were loaded with lipids or lipofuscin granules and clustered mainly in the apical sheath area. The most damaged epithelial cells with lysis areas and intense macrophage activity, highlighted by accumulation of melanosomes and lipofuscin granules, were observed in both sexes but were more often evident in the aged male retina.

**TABLE 1:** Electron microscopy analysis of Bruch’s membrane thickness (nm) of the aged retina.

Gender	Control retina	Aged retina
Male	571 ± 117	702 ± 117^a^
Female	409 ± 54	710 ± 83^a^

^a^ *p* < 0.001 aged retina vs. control (young).

**FIGURE 4:**
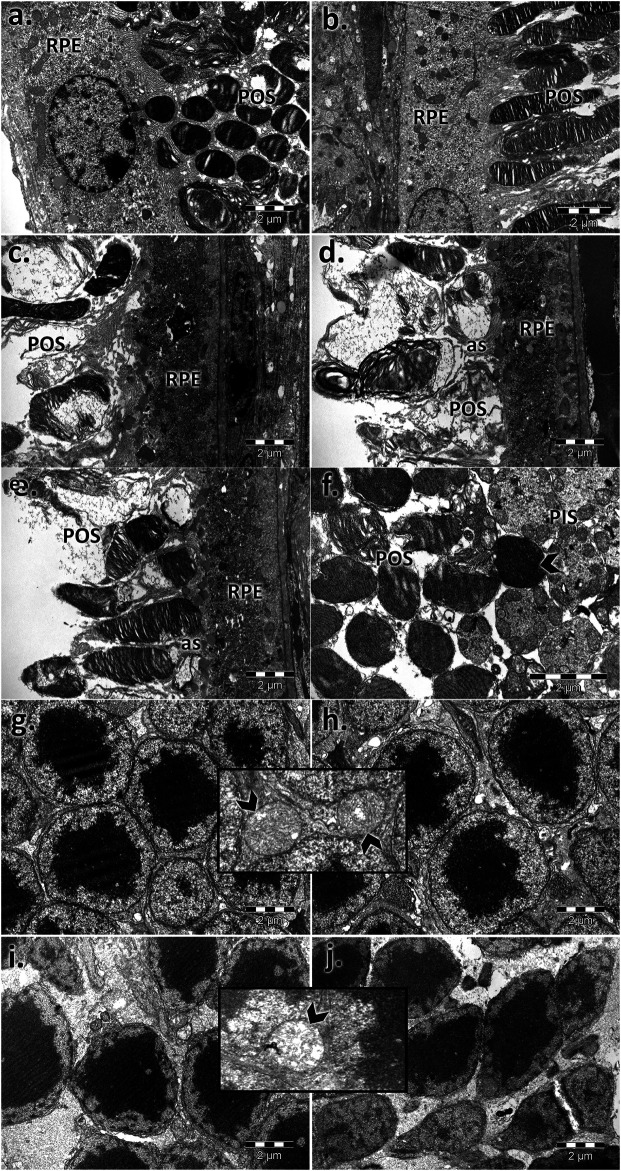
Electron micrographs showing the ultrastructural features of the photoreceptor layer (PS) and outer nuclear layer (ONL) of the young and aged retinas. PS of control male **(A)** and female **(B)** mice; PS of aged male **(C,D)** and female **(E,F)** mice; ONL of control male **(G)** and female **(H)** mice; ONL of aged male **(I)** and female **(J)** mice; photoreceptor outer segments with aligned discs and normal structure **(A,B)**; RPE, retinal pigment epithelial layer; ONL, outer nuclear layer; POS, photoreceptor outer segments; the POS are misoriented, damaged, and inconsistent and accompanied by apical sheaths (as) of the epithelial cells (c, d, and **E)** and condensed **(F)**; photoreceptor outer (POS) and inner segment layer (PIS); normal aspect of the photoreceptor nuclei, aligned and densely packaged **(G and H)** with the normal aspect of the mitochondria (detail—arrowhead); photoreceptor nuclei with different sizes and chromatin density and empty spaces; swelling of mitochondria with a loss of cristae (detail—arrowhead) **(I and J)**. Figures are representative of *n* = 10 electron micrographs for each individual/group.

For both controls, the photoreceptor discs were intact and properly aligned and were perpendicular to the photoreceptor axis. In aging states, impairment of the photoreceptor outer segments was observed (Figure 4). The rod and cone outer segments were surrounded or intermittently surrounded by the apical sheaths of epithelial cells and were randomly oriented, truncated, or absent. Massive vesiculation and fragmentation of outer segment lamellae were observed in both aged retinas but mainly in male samples upon electron microscopy.

Moreover, in old mice, we found photoreceptor nuclei of variable shapes, sizes, and chromatin densities, and some had pyknotic nuclei and empty spaces, indicating nuclear loss. Swelling of mitochondria was observed mainly in aged male photoreceptor cells (Figure 4) compared with similarly processed samples from females.

**FIGURE 5:**
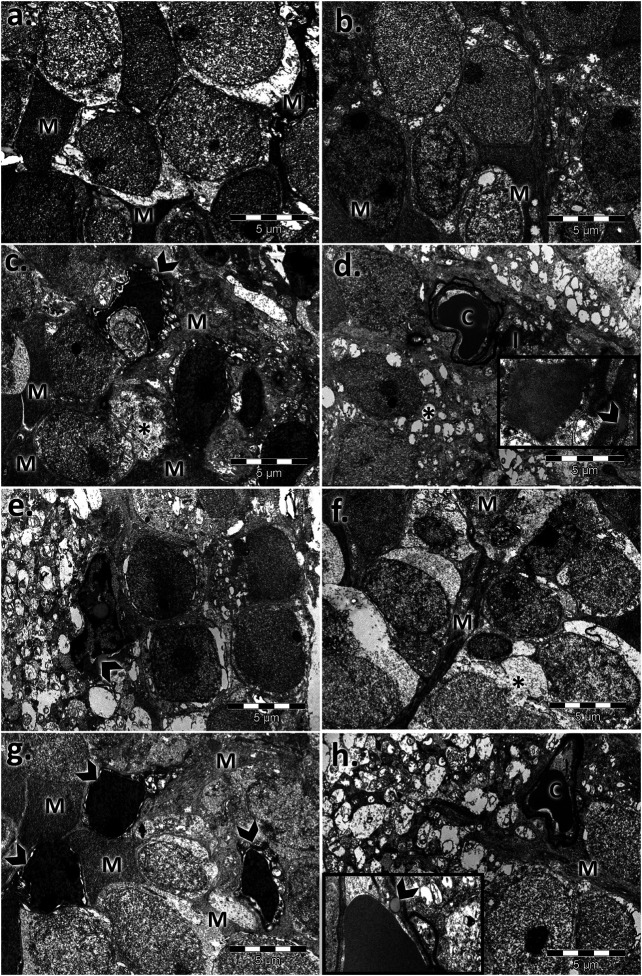
Electron micrographs showing the ultrastructural features of the inner nuclear layer (INL) of the young and aged retinas. **(A)** control male; **(B)** control female; **(C,F)** aged male retina—hypertrophied Müller cells (M), condensed Müller cell nuclei (arrowhead), bipolar cells (B), and capillaries (C) with a thickened basement membrane and lipids (l); empty spaces (*); detail: lipid accumulation beside the capillary and into the basal membrane (arrowhead); damaged INL areas in the aged male retina with microglia (arrowhead) **(G**,**H)** aged female retina—hypertrophied Müller cells (M); condensed Müller cells nuclei (arrowhead), capillary with thickened basement membrane (C); bipolar cells (B); detail: lipid accumulation beside the capillaries and into the basal membrane (arrowhead). Figures are representative of *n* = 10 electron micrographs for each individual/group.

The inner nuclear layer (INL) of the controls had a normal aspect, was abundant in bipolar cells, and ensheathed by processes of Müller cells. Electron micrographs of both aged retinas showed an extensive network of cytoplasmic processes of Müller cell wrapping around other cells and penetrating the neighboring inner plexiform layer (IPL) (Figure 5). Additionally, we registered marked thickening of the endothelial basal membrane of the capillary and lipid accumulation. Microglial hypertrophy was observed in the extended damaged INL areas.

**FIGURE 6:**
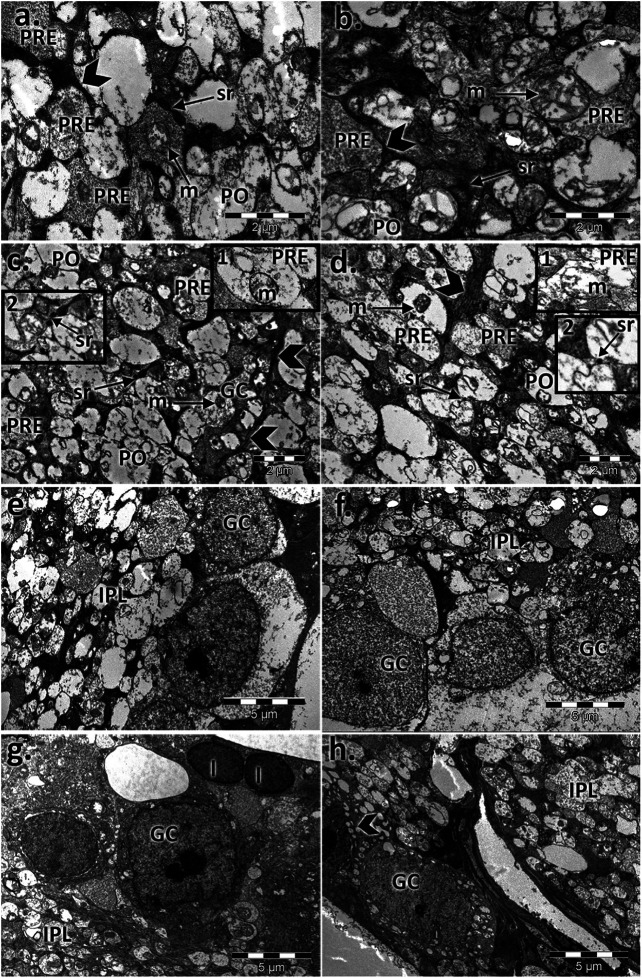
Electron micrographs showing the ultrastructural features of the inner plexiform layer (IPL) and ganglion layer (RGL) of the young and aged retinas. Ultrathin section through the inner plexiform layer (IPL) showing different types of processes in **(A)** the control male, **(B)** control female, **(C)** aged male retina, and **(D)** aged female retina; presynaptic endings (PRE); large mitochondria (m); postsynaptic endings (PO); Müller processes (MP) (arrowhead); detail 1: presynaptic endings (PRE) with rarefied mitochondrial cristae and a few microvesicles—aged males **(C)** and females **(D)**; detail 2: synaptic ribbons (arrows) in a presynaptic bag (PRE)—aged males **(C)** and females **(D)**; electron micrographs of a section through the ganglion cell layer in **(E)** the control male, **(F)** control female **(G)**, aged male retina **(H)**, and aged female retina; ganglion cell (GC), inner plexiform layer (IPL), apoptotic cell (arrowhead), and lipofuscin granules (l). Figures are representative of *n* = 10 electron micrographs for each individual/group.

The inner plexiform layer of the controls clearly showed different types of processes (Figure 6). The presynaptic endings (PRE) were full of microvesicles and contained one or more large mitochondria (m). Postsynaptic endings (PO) also presented mitochondria (m) but very few or no microvesicles. Müller processes (MP) were clear and contained few filaments (arrows). The aged retinas contained atrophied synaptic endings. The main difference was in the presynaptic endings, which were almost deprived by microvesicles, and most of the mitochondria presented rarefied cristae. The Müller processes (MP) were spread into the INL with proliferate filaments (arrows). The synaptic ribbons in the presynaptic bag (PRE) decreased in number and were in a much smaller size than the control for both sexes.

The ganglion layer was also affected with age (Figure 6). The number of ganglion cells decreased, some had apoptotic aspects, and lipofuscin granules were accumulated. Müller cell processes, microglia, and astrocytes surrounded the blood vessels, which possessed a thickened basal membrane.

### Aging-Induced Retinal miRNA Dysregulation in Aged Mouse Retina for Both Sexes

**FIGURE 7:**
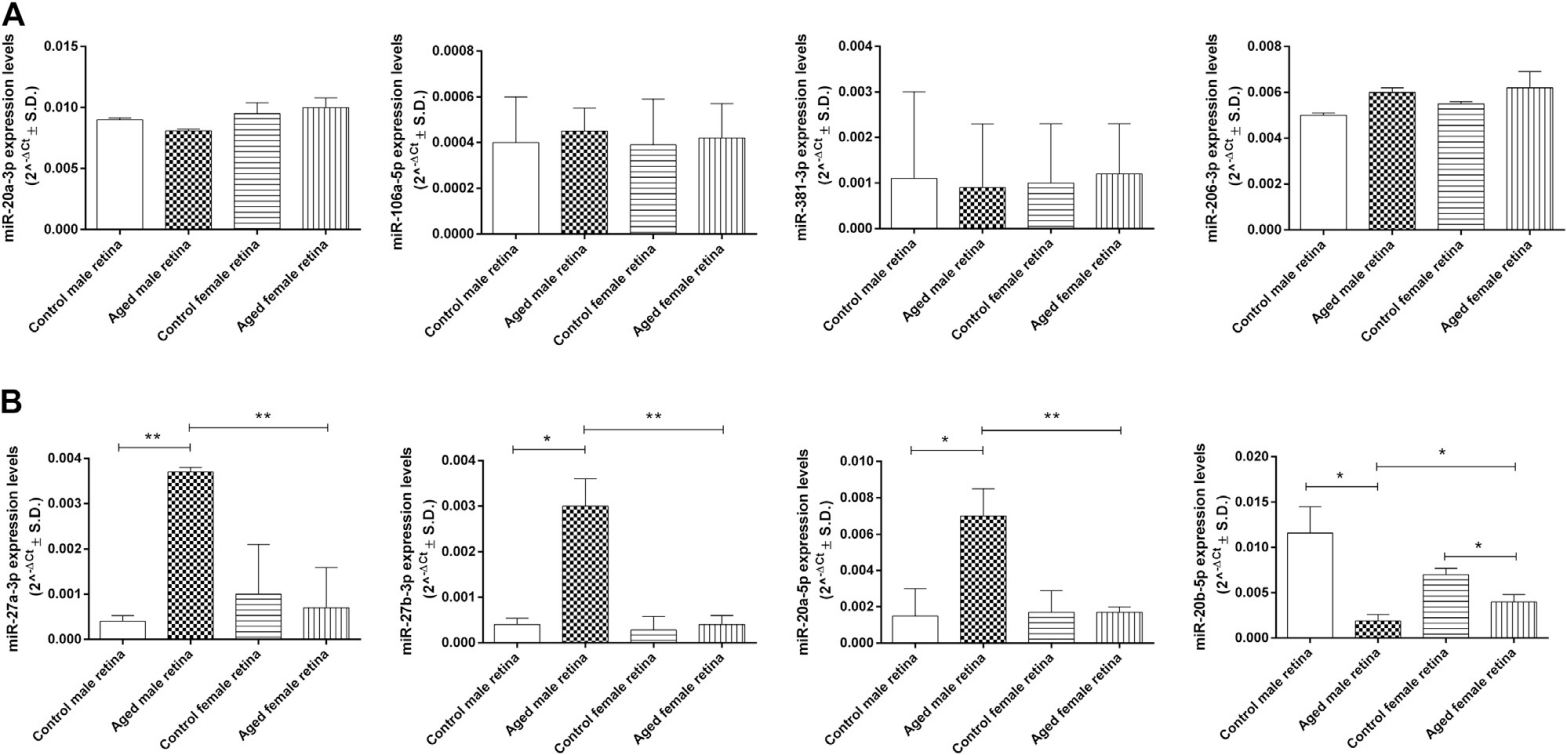
Age-related miRNA expression levels in aged retina. **(A)** Not dysregulated and **(B)** dysregulated retina miRNA expression levels. Data are reported as 2^^−ΔCt^ and shown as mean ± SD of *n* = 10 observations for each experimental group; each run was performed in triplicate. Statistical significance was assessed by using one-way ANOVA, followed by Tukey’s multiple comparisons test. *p < 0.05 and **p < 0.01.

Four of the eight miRNAs analyzed (miR-20a-3p, miR-106a-5p, miR-381-3p, and miR-206-3p) were not dysregulated between the control and aged experimental groups (Figure 7A). Conversely, miR-27a-3p (fold regulation = 8.9; *p* < 0.01), miR-27b-3p (fold regulation = 8.1; *p* < 0.05), and miR-20a-5p (fold regulation = 4.9; *p* < 0.05) were significantly up-regulated in aged male mice compared to control male mice, whereas miR-20b-5p was significantly down-regulated (fold regulation = 3.9; *p* < 0.05) (Figure 7B). Moreover, miR-20b-5p was significantly down-regulated in aged female mice compared to control female mice (fold regulation = 2.5; *p* < 0.05) (Figure 7B). miR-27a-3p was significantly up-regulated in aged males (fold regulation = 5.00, *p* < 0.01) compared to that in aged females as well as miR-27b-3p (fold regulation = 7.58, *p* < 0.01) and miR-20a-5p (fold regulation = 4.11, *p* < 0.01), whereas miR-20b-5p was significantly down-regulated in aged males (fold regulation = −2,10, *p* < 0.05) compared to that in aged females (Figure 7B).

### Correlation between Aged miRNAs and Retinal Structure

**FIGURE 8:**
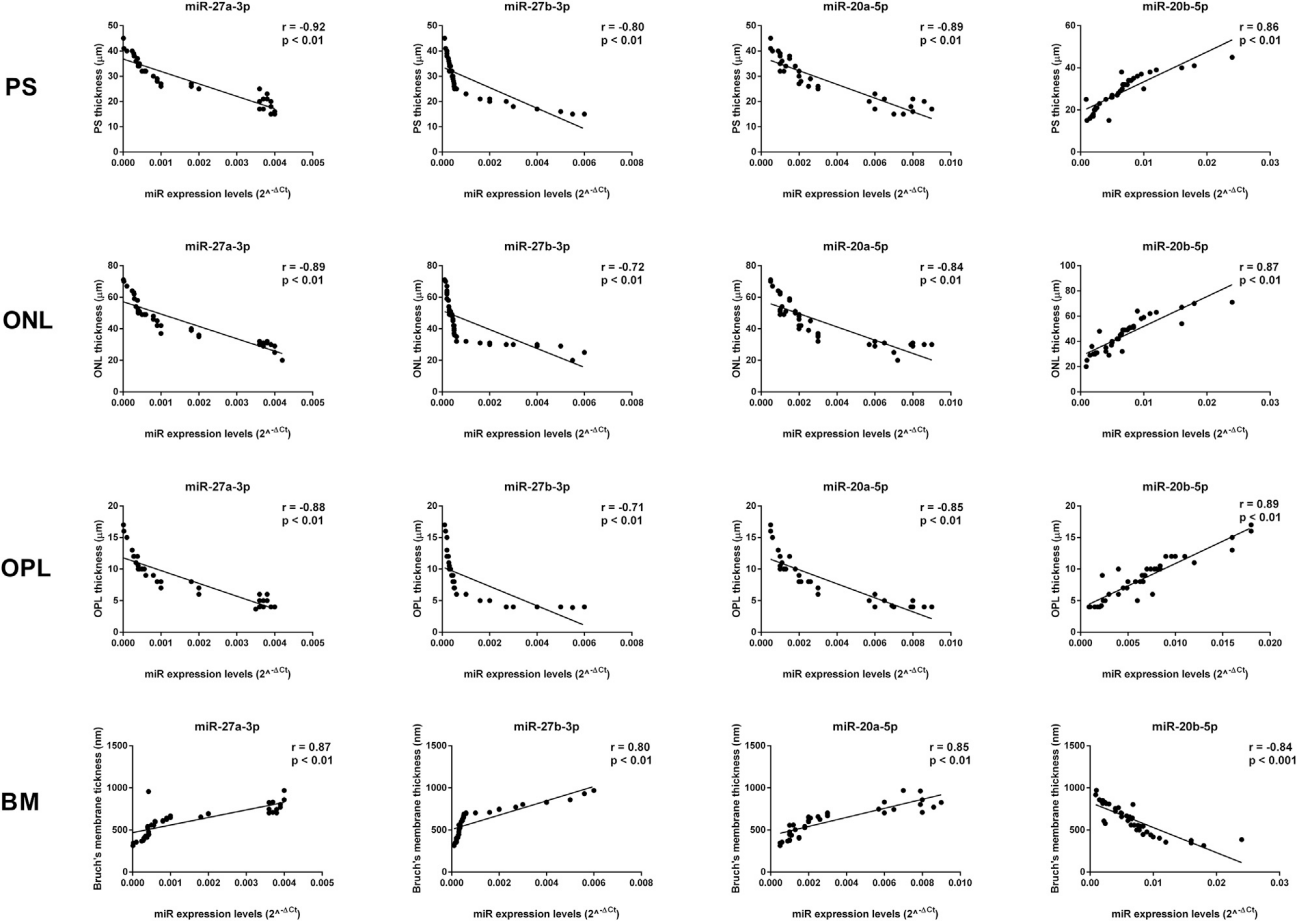
Significant correlation between age-related miRNA expression levels and retinal structure. miR-27a-3p, miR-27b-3p, and miR-20a-5p levels showed a negative correlation with PS, ONL, and OPL thickness, and they showed a positive correlation with Bruch’s membrane thickness. miR-20b-5p was positively correlated with PS, ONL, and OPL thickness, and it was negatively correlated with Bruch’s membrane thickness. Pearson correlation analysis was used to evaluate the strength of association between pairs of variable, by including all the samples with different age and gender. Differences were considered statistically significant for *p* values <0.05. PS, retinal photoreceptors layer; ONL, retinal outer nuclear layer; OPL, retinal outer plexiform layer; BM, Bruch’s membrane.

**FIGURE 9:**
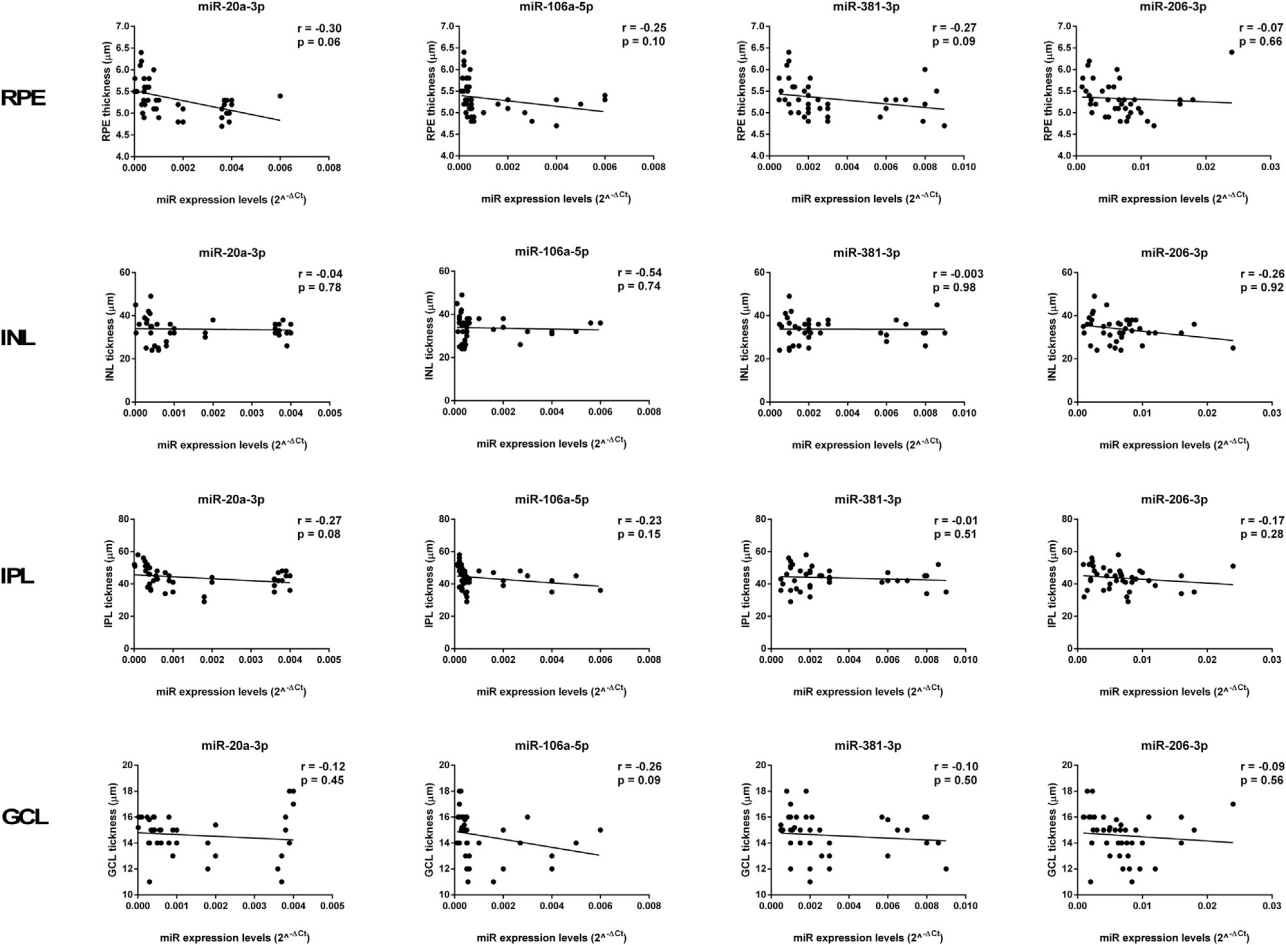
Age-related miRNA expression levels not correlated with retina structure. No significant correlations were observed between RPE, INL, IPL, and GCL thickness and the miR-20a-3p, miR-106a-5p, miR-381-3p, and miR-206-3p expression levels. Pearson correlation analysis was used to evaluate the strength of association between pairs of variables, by including all the samples with different age and gender. Differences were considered statistically significant for *p* values <0.05. RPE, retinal pigment cells; INL, retinal inner nuclear layer; IPL, retinal inner plexiform layer; GCL, retinal ganglion cell layer.

The thickness of the PS, ONL, and OPL negatively correlated with miR-27a-3p, miR-27b-3p, and miR-20a-5p levels (*p* < 0.01), whereas miR-20b-5p expression was positively correlated with PS, ONL, and OPL thickness (*p* < 0.01) (Figures 8, 9). In contrast, miR-27a-3p, miR-27b-3p, and miR-20a-5p levels were positively correlated with Bruch’s membrane thickness (*p* < 0.01), whereas it showed a negative correlation with miR-20b-5p expression (*p* < 0.01) (Figure 9).

## Discussion

Age and gender are two important factors that may influence the function and structure of the retina. Other results did not show a significant difference between the thickness of the retinal layers and age, by sex in groups of Sprague Dawley rats aged 1, 2, 6, and 10 months (Chaychi et al., 2015), which highlights that major structural changes appear only in the second year of life in rodents (approximately 50 years in humans). In our study, the photoreceptors appeared to be more affected with age. This was particularly evident in males. Our results and a study by DiLoreto et al. (1994) showed that the peripheral retinal thickness reduction documented in female CD mice and female F344 rats was delayed and less significant compared to that in males. In addition, an attenuation of ERG amplitudes between 1 and 10 months of age was recorded (Chaychi et al., 2015), which may explain the fine changes in the ocular media and the gradual reduction in photopigment contents (Birch and Anderson, 1992) or the reduction in retinal cell number with age (Dorey et al., 1989; Curcio et al., 1990), as our data showed for both sexes.

Age-related macular degeneration (AMD) in humans is associated with progressive degeneration of the retinal pigment epithelium (RPE) cell layer and photoreceptor cells. Because photoreceptors depend for their maintenance on RPE, it was essential to analyze the involvement of RPE in changes related to aging. The senescence-accelerated mouse (SAM) strain was shown to exhibit changes only in Bruch’s membrane and RPE (Ogata et al., 1992, Takada et al., 1993; Takada et al., 1994). Electron microscopy measurements of Bruch’s membrane of the SAM showed thickening with age, starting with 12 months (Majji et al., 2000), whereas our results in 24-month-old CD1 mice confirmed an increased thickness for both sexes (Table 1), and electron micrographs showed deposits with similar characteristics to the basal laminar material seen in AMD (Green and Enger, 1993). The RPE and Bruch’s membrane changes observed in our study were also reported by several studies (Ogata et al., 1992; Takada et al., 1993, Takada et al., 1994; Nakamura et al., 1995). They showed disorganization of the basal infolding, extension of the intercellular space, and accumulation of lipofuscin granules, which are all in agreement with our results. According to a SAM experiment for mice older than 12 months (Majji et al., 2000), we observed at 24 months of age localized rough extensions in Bruch’s membrane toward the RPE (related to basal linear deposits found in human AMD) and a general increase in the thickness of Bruch’s membrane. Other studies found the presence of drusen between the RPE basal lamina and inner collagenous layer of BM (Farkas et al., 1971; Loffler and Lee, 1986; van der Schaft et al., 1991; van der Schaft et al., 1993; Kliffen et al., 1997; Zarbin, 1998; Curcio and Millican, 1999; Green, 1999; Sarks et al., 1999, 2007; Spraul et al., 1999). Both basal deposits and drusen result from incomplete digestion/removal of RPE cellular constituents (Farkas et al., 1971) and are the main cause of photoreceptor deficiencies, making it difficult to obtain nutrients from the vascular choroid.

Aging is also associated with accumulation of retinal lipids and lipofuscins, subsequently contributing to AMD (Suzuki et al., 2007; Curcio et al., 2009), to best vitelliform macular dystrophy, or to fundus flavimaculatus (Stargardt disease) (Nag and Wadhwa, 2012). In particular, electron micrographs showed RPE lipid accumulation in both aged males and females and infiltrated into Bruch’s membrane. The abundant lipids could contribute to the degeneration of the RPE and subsequent to the photoreceptors, whose metabolism depends on normal epithelial function and layer integrity.

The lipid source of photoreceptors is plasma lipids, and their transfer is facilitated by the RPE and Müller cells. Moreover, the receptors for LDL were highlighted on the RPE (Hayes et al., 1989; Gordiyenko et al., 2004) connected with local production of apoE by RPE cells (Trivino et al., 2006), and a disturbed metabolism could cause an imbalance and lead to intracellular lipid accumulation. The metabolism and transport of lipids could be more impaired as a result of a thickened Bruch’s membrane with changes in its collagen layers and deposition (Pauleikoff et al., 1990; Curcio et al., 2001; Trivino et al., 2006). Curcio et al. (2001) assumed that the aging process of Bruch’s membrane is similar to that of the arterial intima and other connective tissues for which lipoproteins are the source of extracellular cholesterol. The lipid drops observed in Bruch’s membrane for both aged males and females may arise from esterified cholesterol-rich apolipoprotein B–containing lipoprotein particles produced by the RPE (Curcio et al., 2010).

The outer segments of the photoreceptors are composed of infolded plasma membrane discs with photopigments that are properly aligned, and establish an anatomical association with the RPE (Johnson et al., 1986; Blanks et al., 1988). Several studies have shown the misalignment of the outer photoreceptor segments in aging and pathology (as AMD) (Green and Enger, 1993; Jackson et al., 2002; Liu et al., 2003; Eckmiller, 2004), and the discs are randomly oriented or disorganized into tubules and membranous whorls (Marshall et al., 1979; Nag and Wadhwa, 2012), as we observed in the 75- to 85-year-old human-equivalent aged mice (24-month-old mice), mainly for aged males. Their occurrence is possibly attributed to the vulnerability of mitochondria via oxidative stress, light, or toxic substances, contributing to energy depletion and cone loss in the human retina with aging (Curcio and Drucker, 1993). Moreover, injured mitochondria have been reported by us in photoreceptor cells and are more widespread for the aged male retina.

A proposed mechanism that could trigger the degenerative process in aging is primarily focused on the outer segments of the photoreceptors, followed by the inner segments, and finally on the cell body depletion from the ONL (DiLoreto et al., 1994). A deeper investigation of retinal changes with age suggests hormonal involvement in structural and functional preservation, such as estrogen. Chaychi et al. (2015) assumed the role of biological sex and age on the retinal function. They showed better retinal functions in cyclic female rats than in menopausal ones, suggesting a negative effect of the estrus cycle decline related to aging (van den Beld et al., 2018). Other mechanistic involvement in sex difference targets the oxygen supply of the retina. In a recent preclinical experiment, female neurons were reported to be less sensitive than male neurons to hypoxia induced by oxygen–glucose deprivation (Lang and McCullough, 2008). Moreover, estrogen can attenuate retinal ischemia–reperfusion (Nonaka et al., 2000) via nitric oxide and nitric oxide synthase (NOS) activity enhancement genes (Mendelsohn and Karas, 2005).

The INL contain three types of neurons (horizontal, bipolar, and amacrine cells), a macroglia, and a Müller cell, which span the entire thickness of the retina, and they are in anatomical and functional contact with all retinal neurons. The phenotype changes of the Müller glial cells are highlighted by the presence of an extensive network of cytoplasmic processes wrapping around other cells and penetrating the neighboring inner plexiform layer (IPL). The electron microscopy observations may be correlated with data regarding Müller cell resistance in ischemia, anoxia, or hypoglycemia (Silver et al., 1997; Stone et al., 1999) to the detriment of neurons and their passage into the activated or “reactive” state in response to every pathological alteration of the retina (Bringmann et al., 2006). For example, in proliferative vitreoretinopathy (PVR), they undergo hypertrophy (Francke et al., 2001). Moreover, under pathological conditions, they could induce functional changes with microglial activation of the retina (Bringmann et al., 2006), as we pointed out in aging mice. Regarding vascular changes, we showed marked thickening of the endothelial basal membrane of the capillary, which is a change that has been encountered in other pathological conditions, such as AMD (Ramírez et al., 2001) and DR (Ashton, 1974; Garner, 1993; Cai and Boulton, 2002), although the thickening of the endothelial basal membrane of retinal capillaries was recently reported to occur with aging, besides in diseases (Nag et al., 2019).

The ultrastructural aspect of the ganglionar layer was affected with age with a decline in the number of ganglion cells, as was shown by other aging studies (Curcio and Drucker, 1993) or other ocular pathology studies, such as those on glaucoma, optic neuritis, RP, and Alzheimer’s disease (Hinton et al., 1986; Stone et al., 1992; Curcio and Drucker, 1993; Trip et al., 2005; Osborne, 2008). In our electron microscopy investigation, we observed lipofuscin granule accumulation, which prominently occurred in ganglion cells in patients with Batten disease (Bensaoula et al., 2000) but were also observed in other aging experiments (Nag et al., 2006).

MicroRNAs (miRNAs) have been established as significant regulators of senescence and cell aging (Smith-Vikos and Slack, 2012) by inducing mRNA degradation or translational repression (Bartel, 2004). miR-27a target genes are important for glutathione metabolism (Smith-Vikos and Slack, 2012) and are involved in suppression of the expression of the enzymes important for polyamine biosynthesis, such as ornithine decarboxylase (Bates et al., 2010). In our study, we found a significant up-regulation of miR-27a-3p in aged retinas compared to young ones, as well as in aged males compared to aged females. This was paralleled by our electron microscopic observations showing ultrastructural changes segregated by sex, which were specific to aged males. In particular, miR-27a was negatively correlated with the thickness of PS, ONL, and OPS, whereas it was positively correlated with Bruch’s membrane thickness. Of note, glutathione reduction leads to photoreceptor cell death, a reduction in ONL thickness, and increased Bruch’s membrane thickness in accordance with our data (Winkler and Giblin, 1983; Schimel et al., 2011; Fernandez-Robredo et al., 2013). Interestingly, miR-27a-3p and miR-20a-5p up-regulation in aged mice is in line with previous studied that showed an elevation of these miRNAs in the serum and plasma of patients affected by wet or dry age-related macular degeneration (AMD), an ocular pathology characterized by cellular debris between the choroid and retina (Ertekin et al., 2014; Berber et al., 2017; Romano et al., 2017). miR-27b-3p, an oxidative stress-responsive microRNA, was found to be up-regulated in aged mice and previously showed significant age differences in the male mice thymus (Guo et al., 2017; Xu et al., 2017). Moreover, miR-20b-5p, which was found to be down-regulated by oxidative stress (Tai et al., 2020), was one of the most important causes underlying oxidative damage during aging (Liguori et al., 2018), and it was reported to inhibit the senescence of human umbilical vein endothelial cells induced by oxidative stress by promoting cell viability (Dong et al., 2020).

Further evidence supporting a dysregulation of age-related miRNAs in the retinal structure and function was shown by our correlation analysis, showing a significant association between miRNA expression levels and the thicknesses of PS, ONL, OPL, and Bruch’s membrane. Although miR-27a-3p, miR-27b-3p, miR-20a-5p, and miR-20b-5p were shown to be dysregulated in retinal derangement induced by diabetes (Platania et al., 2019), these miRNAs were here strictly correlated with the alterations of the retina induced during aging. Indeed, although gender-related miRNA expression remains largely unexplored, with few studies showing a higher tissue specificity associated with histone modification and circadian rhythm for male-biased miRNAs and a higher disease spectrum associated with metabolism and cell cycle process for female-biased miRNA (Cui et al., 2018), our results showed that biological sex can influence the structure and function of the retina upon aging, suggesting that this difference may be underlined by the dysregulation of age-related mi-RNAs.

Interestingly, gender-related miRNAs have been previously reported to be dysregulated in neurological disorders: miR-27a was up-regulated in epilepsy and autoimmune encephalomyelitis (Wang et al., 2014; Morquette et al., 2019); higher levels of miR-27b exacerbate intracerebral hemorrhage-induced brain injury (Xu et al., 2017) and are related to the development of neurological disorders with HCMV infection (Wang et al., 2017); miR-20a showed an increased expression in traumatic injury (Wang et al., 2014); and miR-20b was found to be down-regulated in multiple sclerosis (Ingwersen et al., 2015). Although there is no evidence regarding the gender-related expression of miR-27a, miR-27b, miR-20a, and miR-20b in these neurological disorders, miR-20b expression was higher in males during aging of the thymus (Guo et al., 2017); miR-20a-5p has recently been found to be associated with male infertility (Cito et al., 2020), whereas miR-27a has been found to be related to endometritis (Di Pietro et al., 2018). The expression of mir-20a and miR-20b was highest in the male lung, whereas miR-27b was more expressed in the female lung (Cui et al., 2018). Therefore, studying the gender-related retinal morphological differences and the dysregulation of some retinal miRNAs upon aging, which are closely related to the functional alterations and the development of subsequent pathologies, may provide insights into the neuroprotective mechanisms. From these age-related damaging mechanisms, the increased susceptibility of Balb-c mice to light damage seems to be excluded because one would expect to observe higher retinal morphological damage in young animals than in aged animals (Kalesnykas et al., 2016).

## Data Availability

The raw data supporting the conclusions of this article will be made available by the authors, without undue reservation.

## References

[B1] Adhi M. Aziz S. Muhammad K. Adhi M. I. (2012). Macular thickness by age and gender in healthy eyes using spectral domain optical coherence tomography. PLoS One. 7, e37638. 10.1371/journal.pone.0037638 22629435 PMC3357395

[B2] Age-Related Eye Disease Study Research Group (2000). Risk factors associated with age-related macular degeneration. A case-control study in the age-related eye disease study: age-Related Eye Disease Study Report Number 3. Ophthalmology. 107, 2224–2232. 10.1016/s0161-6420(00)00409-7 11097601 PMC1470467

[B3] Ashton N. (1974). Vascular basement membrane changes in diabetic retinopathy. Montgomery lecture, 1973. Br. J. Ophthalmol. 58, 344–366. 10.1136/bjo.58.4.344 4138036 PMC1214780

[B4] Bartel D. P. (2004). MicroRNAs: genomics, biogenesis, mechanism, and function. Cell. 116, 281–297. 10.1016/s0092-8674(04)00045-5 14744438

[B5] Bates D. J. Li N. Liang R. Sarojini H. An J. Masternak M. M. (2010). MicroRNA regulation in Ames dwarf mouse liver may contribute to delayed aging. Aging Cell. 9, 1–18. 10.1111/j.1474-9726.2009.00529.x 19878148 PMC2844644

[B6] Beatty S. Koh H. Phil M. Henson D. Boulton M. (2000). The role of oxidative stress in the pathogenesis of age-related macular degeneration. Surv. Ophthalmol. 45, 115–134. 10.1016/s0039-6257(00)00140-5 11033038

[B7] Beebe D. C. Holekamp N. M. Shui Y. B. (2010). Oxidative damage and the prevention of age-related cataracts. Ophthalmic Res. 44, 155–165. 10.1159/000316481 20829639 PMC2952186

[B8] Bell B. A. Kaul C. Bonilha V. L. Rayborn M. E. Shadrach K. Hollyfield J. G. (2015). The BALB/c mouse: effect of standard vivarium lighting on retinal pathology during aging. Exp. Eye Res. 135, 192–205. 10.1016/j.exer.2015.04.009 25895728 PMC4446204

[B9] Bensaoula T. Shibuya H. Katz M. L. Smith J. E. Johnson G. S. John S. K. (2000). Histopathologic and immunocytochemical analysis of the retina and ocular tissues in Batten disease. Ophthalmology. 107, 1746–1753. 10.1016/s0161-6420(00)00264-5 10964839

[B10] Berber P. Grassmann F. Kiel C. Weber B. H. (2017). An eye on age-related macular degeneration: the role of microRNAs in disease pathology. Mol. Diagn. Ther. 21, 31–43. 10.1007/s40291-016-0234-z 27658786 PMC5250647

[B11] Birch D. G. Anderson J. L. (1992). Standardized full-field electroretinography. Normal values and their variation with age. Arch. Ophthalmol. 110, 1571–1576. 10.1001/archopht.1992.01080230071024 1444914

[B12] Blanks J. C. Hageman G. S. Johnson L. V. Spee C. (1988). Ultrastructural visualization of primate cone photoreceptor matrix sheaths. J. Comp. Neurol. 270, 288–300. 10.1002/cne.902700209 3379160

[B13] Bozycki L. Łukasiewicz K. Matryba P. Pikula S. (2018). Whole-body clearing, staining and screening of calcium deposits in the mdx mouse model of Duchenne muscular dystrophy. Skelet. Muscle. 8, 21. 10.1186/s13395-018-0168-8 30025544 PMC6053777

[B14] Bringmann A. Pannicke T. Grosche J. Francke M. Wiedemann P. Skatchkov S. N. (2006). Müller cells in the healthy and diseased retina. Prog. Retin. Eye Res. 25, 397–424. 10.1016/j.preteyeres.2006.05.003 16839797

[B15] Brûlé J. Lavoie M. P. Casanova C. Lachapelle P. Hébert M. (2007). Evidence of a possible impact of the menstrual cycle on the reproducibility of scotopic ERGs in women. Doc. Ophthalmol. 114, 125–134. 10.1007/s10633-007-9045-1 17273847

[B16] Buch H. Nielsen N. V. Vinding T. Jensen G. B. Prause J. U. la Cour M. (2005). 14-year incidence, progression, and visual morbidity of age-related maculopathy: the Copenhagen City Eye Study. Ophthalmology. 112, 787–798. 10.1016/j.ophtha.2004.11.040 15878058

[B17] Cai J. Boulton M. (2002). The pathogenesis of diabetic retinopathy: old concepts and new questions. Eye. 16, 242–260. 10.1038/sj.eye.6700133 12032713

[B18] Chakravarthy U. Wong T. Y. Fletcher A. Piault E. Evans C. Zlateva G. (2010). Clinical risk factors for age-related macular degeneration: a systematic review and meta-analysis. BMC Ophthalmol. 10, 31. 10.1186/1471-2415-10-31 21144031 PMC3009619

[B19] Chaychi S. Polosa A. Lachapelle P. (2015). Differences in retinal structure and function between aging male and female Sprague-Dawley rats are strongly influenced by the estrus cycle. PLoS One. 10, e0136056. 10.1371/journal.pone.0136056 26317201 PMC4552560

[B20] Cito G. Coccia M. E. Salvianti F. Fucci R. Picone R. Giachini C. (2020). Blood plasma miR-20a-5p expression as a potential non-invasive diagnostic biomarker of male infertility: a pilot study. Andrology. 8, 1256–1264. 10.1111/andr.12816 32406197

[B21] Cui C. Yang W. Shi J. Zhou Y. Yang J. Cui Q. (2018). Identification and analysis of human sex-biased microRNAs. Dev. Reprod. Biol. 16, 200–211. 10.1016/j.gpb.2018.03.004 PMC6076379 30005964

[B22] Curcio C. A. Drucker D. N. (1993). Retinal ganglion cells in Alzheimer’s disease and aging. Ann. Neurol. 33, 248–257. 10.1002/ana.410330305 8498808

[B23] Curcio C. A. Johnson M. Huang J. D. Rudolf M. (2009). Aging, age-related macular degeneration, and the response-to-retention of apolipoprotein B-containing lipoproteins. Prog. Retin. Eye Res. 28, 393–422. 10.1016/j.preteyeres.2009.08.001 19698799 PMC4319375

[B24] Curcio C. A. Johnson M. Huang J. D. Rudolf M. (2010). Apolipoprotein B-containing lipoproteins in retinal aging and age-related macular degeneration. J. Lipid Res. 51, 451–467. 10.1194/jlr.R002238 19797256 PMC2817575

[B25] Curcio C. A. Millican C. L. Bailey T. Kruth H. S. (2001) Accumulation of cholesterol with age in human Bruch’s membrane, Invest. Ophthalmol. Vis. Sci. 42, 265–274. 11133878

[B26] Curcio C. A. Millican C. L. (1999). Basal linear deposit and large drusen are specific for early age-related maculopathy. Arch. Ophthalmol. 117, 329–339. 10.1001/archopht.117.3.329 10088810

[B27] Curcio C. Allen K. Kalina R. (1990). Reorganization of the human photoreceptor mosaic following age-related rod loss. Invest. Ophthalmol. Vis. Sci. 31, 38–48.

[B28] Davis M. D. Fisher M. R. Gangnon R. E. Barton F. Aiello L. M. Chew E. Y. (1998). Risk factors for high-risk proliferative diabetic retinopathy and severe visual loss: early treatment diabetic retinopathy study report #18. Invest. Ophthalmol. Vis. Sci. 39, 233–252. 9477980

[B29] Di Pietro C. Caruso S. Battaglia R. Iraci Sareri M. La Ferlita A. Strino F. (2018). MiR-27a-3p and miR-124-3p, upregulated in endometrium and serum from women affected by Chronic Endometritis, are new potential molecular markers of endometrial receptivity. Am. J. Reprod. Immunol. 80, e12858. 10.1111/aji.12858 29663566

[B30] DiLoreto D. Jr. Cox C. Grover D. A. Lazar E. Cerro C. D. Cerro M. D. (1994). The influences of age, retinal topography, and gender on retinal degeneration in the Fischer 344 rat. Brain Res. 647, 181–191. 10.1016/0006-8993(94)91316-1 7922494

[B31] Dong F. Dong S. Liang Y. Wang K. Qin Y. Zhao X. (2020). miR-20b inhibits the senescence of human umbilical vein endothelial cells through regulating the Wnt/β‑catenin pathway via the TXNIP/NLRP3 axis. Int. J. Mol. Med. 45, 847–857. 10.3892/ijmm.2020.4457 31922218 PMC7015131

[B32] Dorey C. K. Wu G. Ebenstein D. Garsd A. Weiter J. (1989). Cell loss in the aging retina. Relationship to lipofuscin accumulation and macular degeneration. Invest. Ophthalmol. Vis. Sci. 30, 1691–1699. 2759786

[B33] Du M. Mangold C. A. Bixler G. V. Brucklacher R. M. Masser D. R. Stout M. B. (2017). Retinal gene expression responses to aging are sexually divergent. Mol. Vis. 152, 707–717. PMC5640516 29062222

[B34] Dutta S. Sengupta P. (2016). Men and mice: relating their ages. Life Sci. 152, 244–248. 10.1016/j.lfs.2015.10.025 26596563

[B35] Eckmiller M. S. (2004). Defective cone photoreceptor cytoskeleton, alignment, feedback, and energetics can lead to energy depletion in macular degeneration. Prog. Retin. Eye Res. 23, 495–522. 10.1016/j.preteyeres.2004.04.005 15302348

[B36] Ertekin S. Yıldırım O. Dinç E. Ayaz L. Fidancı S. B. Tamer L., (2014). Evaluation of circulating miRNAs in wet age-related macular degeneration. Mol. Vis. 20, 1057. 25221421 PMC4113960

[B37] Farkas T. Sylvester V. Archer D. (1971). The ultrastructure of drusen. Am. J. Ophthalmol. 71, 1196–1205. 10.1016/0002-9394(71)90963-9 5091118

[B38] Fernandez-Robredo P. Sádaba L. M. Salinas-Alamán A. Recalde S. Rodríguez J. A. García-Layana A. (2013). Effect of lutein and antioxidant supplementation on VEGF expression, MMP-2 activity, and ultrastructural alterations in apolipoprotein E-deficient mouse. Oxid. Med. Cell Longev. 2013, 213505. 10.1155/2013/213505 23738034 PMC3657460

[B39] Francke M. Faude F. Pannicke T. Bringmann A. Eckstein P. Reichelt W. (2001). Electrophysiology of rabbit Müller (glial) cells in experimental retinal detachment and PVR. Invest. Ophthalmol. Vis. Sci. 42, 1072–1079. 11274088

[B40] Freeman E. E. Munoz B. Schein O. D. West S. K. (2001). Hormone replacement therapy and lens opacities: the Salisbury Eye Evaluation project. Arch. Ophthalmol. 119, 1687–1692. 10.1001/archopht.119.11.1687 11709021

[B41] Freund P. R. Watson J. Gilmour G. S. Gaillard F. Sauvé Y. (2011). Differential changes in retina function with normal aging in humans. Doc. Ophthalmol. 122, 177–190. 10.1007/s10633-011-9273-2 21562738

[B42] Garner A. (1993). Histopathology of diabetic retinopathy in man. Eye. 7 (Pt 2), 250–253. 10.1038/eye.1993.58 7607344

[B43] Gordiyenko N. Campos M. Lee J. W. Fariss R. N. Sztein J. Rodriguez I. R. (2004). RPE cells internalize low-density lipoprotein (LDL) and oxidized LDL (oxLDL) in large quantities *in vitro* and *in vivo* . Invest. Ophthalmol. Vis. Sci. 45, 2822–2829. 10.1167/iovs.04-0074 15277509

[B44] Grassmann F. Schoenberger P. G. Brandl C. Schick T. Hasler D. Meister G. (2014). A circulating MicroRNA profile is associated with late-stage neovascular age-related macular degeneration. PLoS One. 9, e107461. 10.1371/journal.pone.0107461 25203061 PMC4159338

[B45] GreenWeber W. R. Enger C. (1993). Age-related macular degeneration histopathologic studies. The 1992 Lorenz E. Zimmerman Lecture. Ophthalmology. 100, 1519–1535. 10.1016/s0161-6420(93)31466-1 7692366

[B46] Green W. R. (1999) Histopathology of age-related macular degeneration, Mol. Vis. 5, 27, 27. 10562651

[B47] Grossniklaus H. E. Nickerson J. M. Edelhauser H. F. Bergman L. A. Berglin L. (2013). Anatomic alterations in aging and age-related diseases of the eye. Invest. Ophthalmol. Vis. Sci. 54, ORSF23–ORSF27. 10.1167/iovs.13-12711 24335063 PMC3864374

[B48] Guo D. Ye Y. Qi J. Tan X. Zhang Y. Ma Y. (2017). Age and sex differences in microRNAs expression during the process of thymus aging. Acta Biochim. Biophys. Sin. 49, 409–419. 10.1093/abbs/gmx029 28369179

[B49] HashamaniLi N. Hashamani S. Murad A. Shah S. M. M. Hashamani M. (2018). Assessing reproducibility and the effects of demographic variables on the normal macular layers using the Spectralis SD-OCT. Clin. Ophthalmol. 12, 1433–1440. 10.2147/OPTH.S172109 30147295 PMC6095115

[B50] Hayes K. C. Lindsey S. Stephan Z. F. Brecker D. (1989). Retinal pigment epithelium possesses both LDL and scavenger receptor activity. Invest. Ophthalmol. Vis. Sci. 30, 225–232. 10.1006/bbrc.2002.6756 2536645

[B51] Hinton D. R. Sadun A. A. Blanks J. C. Miller C. A. (1986). Optic-nerve degeneration in Alzheimer’s disease. N. Engl. J. Med. 315, 485–487. 10.1056/NEJM198608213150804 3736630

[B52] Ingwersen J. Menge T. Wingerath B. Kaya D. Graf J. Prozorovski T. (2015). Natalizumab restores aberrant miRNA expression profile in multiple sclerosis and reveals a critical role for miR-20b. Ann. Clin. Transl. Neurol. 2, 43–55. 10.1002/acn3.152 25642434 PMC4301674

[B53] Jackson G. R. Owsley C. Curcio C. A. (2002). Photoreceptor degeneration and dysfunction in aging and age-related maculopathy. Ageing Res. Rev. 1, 381–396. 10.1016/s1568-1637(02)00007-7 12067593

[B54] Johnson L. V. Hageman G. S. Blanks J. C. (1986). Interphotoreceptor matrix domains ensheath vertebrate cone photoreceptor cells. Invest. Ophthalmol. Vis. Sci. 27, 129–135. 3080382

[B55] Kalesnykas G. Ragauskas S. Kaja S. Tanila H. Leinonen H. O. (2016). Age-related differences in light sensitivity in BALB/c mice. Investig. Ophthalmol. Vis. Sci. 57, 2004.

[B56] Klein B. E. Klein R. Ritter L. L. (1994). Is there evidence of an estrogen effect on age-related lens opacities? The Beaver Dam Eye study. Arch. Ophthalmol. 112, 85–91. 10.1001/archopht.1994.01090130095025 8285900

[B57] Kliffen M. van der Schaft T. L. Mooy C. M. de Jong P. T. (1997). Morphologic changes in age-related maculopathy. Microsc. Res. Tech. 36, 106–122. 10.1002/(sici)1097-0029(19970115)36:2<106:aid-jemt4>3.0.co;2-n 9015257

[B58] Lai K. Cui J. Ni S. Zhang Y. He J. Yao K. (2013). The effects of postmenopausal hormone use on cataract: a meta-analysis. PLoS One. 8, e78647. 10.1371/journal.pone.0078647 24205286 PMC3813478

[B59] Laitinen A. Laatikainen L. Härkänen T. Koskinen S. Reunanen A. (2010). Prevalence of major eye diseases and causes of visual impairment in the adult Finnish population: a nationwide population-based survey. Acta Ophthalmol. 88, 463–471. 10.1111/j.1755-3768.2009.01566.x 19878108

[B60] Lang J. T. McCullough L. D. (2008). Pathways to ischemic neuronal cell death: are sex differences relevant? J. Transl. Med. 6, 33. 10.1186/1479-5876-6-33 18573200 PMC2459157

[B116] Liguori I. Russo G. Curcio F. Bulli G. Aran L. Della‐Morte D. (2018). Oxidative stress, aging, and diseases. Clin. Interv. 13, 757–72. 10.2147/CIA.S158513 PMC5927356 29731617

[B61] Liu Q. Lyubarsky A. Skalet J. H. Pugh E. N. Jr. Pierce E. A. (2003). RP1 is required for the correct stacking of outer segment discs. Invest. Ophthalmol. Vis. Sci. 44, 4171–4183. 10.1167/iovs.03-0410 14507858 PMC1904498

[B62] Loffler K. U. Lee W. R. (1986). Basal linear deposit in the human macula. Graefes Arch. Clin. Exp. Ophthalmol. 224, 493–501. 10.1007/BF02154735 3792844

[B63] Majji A. B. Cao J. Chang K. Y. Hayashi A. Aggarwal S. Grebe R. R. (2000). Age-related retinal pigment epithelium and Bruch’s membrane degeneration in senescence-accelerated mouse. Invest. Ophthalmol. Vis. Sci. 41, 3936–3942. 11053297

[B64] Marshall J. Grindle J. Ansell P. Borwein B. (1979). Convolution in human rods: an ageing process. Br. J. Ophthalmol. 63, 181–187. 10.1136/bjo.63.3.181 435430 PMC1043437

[B65] Menard C. Rezende F. A. Miloudi K. Wilson A. Tétreault N. (2016). MicroRNA signatures in vitreous humour and plasma of patients with exudative AMD. Oncotarget. 7, 19171–19184. 10.18632/oncotarget.8280 27015561 PMC4991373

[B66] Mendelsohn M. E. Karas R. H. (2005). Molecular and cellular basis of cardiovascular gender differences. Science. 308, 1583–1587. 10.1126/science.1112062 15947175

[B67] Meyers K. J. Johnson E. J. Bernstein P. S. Iyengar S. K. Engelman C. D. Karki C. K. (2013). Genetic determinants of macular pigments in women of the carotenoids in age-related eye disease study. Invest. Ophthalmol. Vis. Sci. 54, 2333–2345. 10.1167/iovs.12-10867 23404124 PMC3626525

[B68] Morquette B. Juźwik C. A. Drake S. S. Charabati M. Zhang Y. Lécuyer M. A. (2019). MicroRNA-223 protects neurons from degeneration in experimental autoimmune encephalomyelitis. Brain. 142, 2979–2995. 10.1093/brain/awz245 31412103

[B69] Nag T. C. Maurya M. Roy T. S. (2019). Age-related changes of the human retinal vessels: possible involvement of lipid peroxidation. Ann. Anat. 226, 35–47. 10.1016/j.aanat.2019.06.007 31330304

[B70] Nag T. C. Wadhwa S. Chaudhury S. (2006). The occurrence of cone inclusions in the ageing human retina and their possible effect upon vision: an electron microscope study. Brain Res. Bull. 71, 224–232. 10.1016/j.brainresbull.2006.09.007 17113950

[B71] Nag T. C. Wadhwa S. (2012). Ultrastructure of the human retina in aging and various pathological states. Micron. 43, 759–781. 10.1016/j.micron.2012.01.011 22445096

[B72] Nagasawa H. Mori T. Yanai R. Bern H. A. Mills K. T. (1978). Long-term effects of neonatal hormonal treatments on plasma prolactin levels in female BALB/cfC3H and BALB/c mice. Cancer Res. 38, 942–945. 639048

[B73] Nakamura S. Akiguchi I. Seriu N. Ohnishi K. Takemura M. Ueno M. (1995). Monoamine oxidase-B-positive granular structures in the hippocampus of aged senescence-accelerated mouse (SAMP8). Acta Neuropathol. 90, 626–632. 10.1007/BF00318576 8615084

[B74] National Research Council (2011). Committee for the update of the Guide for the care and use of laboratory animals (2011). Guide for the care and use of laboratory animals. 8th Edn. Washington, DC: National Academies Press. 10.17226/12910

[B75] Nonaka A. Kiryu J. Tsujikawa A. Yamashiro K. Miyamoto K. Nishiwaki H. (2000). Administration of 17beta-estradiol attenuates retinal ischemia-reperfusion injury in rats. Invest. Ophthalmol. Vis. Sci. 41, 2689–2696. 10937584

[B76] Nuzzi R. Scalabrin S. Becco A. Panzica G. (2018). Gonadal hormones and retinal disorders: a review. Front. Endocrinol. 9, 66. 10.3389/fendo.2018.00066 PMC5840201 29551993

[B77] Ogata N. Ohkuma H. Kanai K. Nango K. Takada Y. Uyama M. (1992). Histological changes in the retinal pigment epithelium and Bruch’s membrane in senescence accelerated mouse. Nippon. Ganka Gakkai Zasshi. 96, 180–189. 1558013

[B78] Ooto S. Hangai M. Tomidokoro A. Saito H. Araie M. Otani T. (2011). Effects of age, sex, and axial length on the three-dimensional profile of normal macular layer structures. Invest. Ophthalmol. Vis. Sci. 52, 8769–8779. 10.1167/iovs.11-8388 21989721

[B79] Osborne N. N. (2008). Pathogenesis of ganglion "cell death" in glaucoma and neuroprotection: focus on ganglion cell axonal mitochondria. Prog. Brain Res. 173, 339–352. 10.1016/S0079-6123(08)01124-2 18929120

[B80] Ozawa G. Y. Bearse M. A. Jr. Adams A. J. (2015). Male-female differences in diabetic retinopathy? Curr. Eye Res. 40, 234–246. 10.3109/02713683.2014.958500 25545999

[B81] Ozawa G. Y. Bearse M. A. Jr. Bronson-Castain K. W. Harrison W. W. Schneck M. E. Barez S. (2012). Neurodegenerative differences in the retinas of male and female patients with type 2 diabetes. Invest. Ophthalmol. Vis. Sci. 53, 3040–3046. 10.1167/iovs.11-8226 22491405 PMC3378087

[B82] Pauleikoff D. Harper C. A. Marshall J. Bird A. C. (1990). Aging changes in Bruch’s membrane. A histochemical and morphologic study. Ophthalmology. 97, 171–178. 10.1016/s0161-6420(90)32619-2 1691475

[B83] Platania C. B. M. Maisto R. Trotta M. C. D’Amico M. Rossi S. Gesualdo C. (2019). Retinal and circulating miRNA expression patterns in diabetic retinopathy: an in silico and *in vivo* approach. Br. J. Pharmacol. 176, 2179–2194. 10.1111/bph.14665 30883703 PMC6555853

[B84] Ramírez J. M. Ramírez A. I. Salazar J. J. de Hoz R. Trivino A. (2001). Changes of astrocytes in retinal ageing and age-related macular degeneration. Exp. Eye Res. 73, 601–615. 10.1006/exer.2001.1061 11747361

[B85] Romano G. L. Platania C. B. M. Drago F. Salomone S. Ragusa M. Barbagallo C. (2017). Retinal and circulating miRNAs in age-related macular degeneration: an *in vivo* animal and human study. Front. Pharmacol. 8, 168. 10.3389/fphar.2017.00168 28424619 PMC5371655

[B86] Rossi S. Maisto R. Gesualdo C. Trotta M. C. Ferraraccio F. Kaneva M. K. (2016). Activation of melanocortin receptors MC 1 and MC 5 attenuates retinal damage in experimental diabetic retinopathy. Mediat. Inflamm. 2016, 7368389. 10.1155/2016/7368389 PMC4753692 26949291

[B87] Sarks S. Cherepanoff S. Killingsworth M. Sarks J. (2007). Relationship of basal laminar deposit and membranous debris to the clinical presentation of early age-related macular degeneration. Invest. Ophthalmol. Vis. Sci. 48, 968–977. 10.1167/iovs.06-0443 17325134

[B88] Sarks S. H. Arnold J. J. Killingsworth M. C. Sarks J. P. (1999). Early drusen formation in the normal and aging eye and their relation to age related maculopathy: a clinicopathological study. Br. J. Ophthalmol. 83, 358–368. 10.1136/bjo.83.3.358 10365048 PMC1722952

[B89] Schimel A. M. Abraham L. Cox D. Sene A. Kraus C. Dace D. S. (2011). N-acetylcysteine amide (NACA) prevents retinal degeneration by up-regulating reduced glutathione production and reversing lipid peroxidation. Am. J. Pathol. 178, 2032–2043. 10.1016/j.ajpath.2011.01.036 21457933 PMC3081196

[B90] Schmidl D. Schmetterer L. Garhöfer G. Popa-Cherecheanu A. (2015). Gender differences in ocular blood flow. Curr. Eye Res. 40, 201–212. 10.3109/02713683.2014.906625 24892919 PMC4364251

[B91] Silver I. A. Deas J. Erecińska M. (1997). Ion homeostasis in brain cells: differences in intracellular ion responses to energy limitation between cultured neurons and glial cells. Neuroscience. 78, 589–601. 10.1016/s0306-4522(96)00600-8 9145812

[B92] Smith-Vikos T. Slack F. J. (2012). MicroRNAs and their roles in aging. J. Cell Sci. 125, 7–17. 10.1242/jcs.099200 22294612 PMC3269020

[B93] Spraul C. W. Lang G. E. Grossniklaus H. E. Lang G. K. (1999). Histologic and morphometric analysis of the choroid, Bruch’s membrane, and retinal pigment epithelium in postmortem eyes with age-related macular degeneration and histologic examination of surgically excised choroidal neovascular membranes. Surv. Ophthalmol. 44 Suppl 1, S10–S32. 10.1016/s0039-6257(99)00086-7 10548114

[B94] Stone E. M. Newman N. J. Miller N. R. Johns D. R. Lott M. T. Wallace D. C. (1992). Visual recovery in patients with Leber’s hereditary optic neuropathy and the 11778 mutation. J. Clin. Neuro Ophthalmol. 12, 10–14. 1532593

[B95] Stone J. Maslim J. Valter-Kocsi K. Mervin K. Bowers F. Chu Y. (1999). Mechanisms of photoreceptor death and survival in mammalian retina. Prog. Retin. Eye Res. 18, 689–735. 10.1016/s1350-9462(98)00032-9 10530749

[B96] Suzuki M. Kamei M. Itabe H. Yoneda K. Bando H. Kume N. (2007). Oxidized phospholipids in the macula increase with age and in eyes with age-related macular degeneration. Mol. Vis. 13, 772–778. 17563727 PMC2768762

[B97] Szemraj M. Bielecka-Kowalska A. Oszajca K. Krajewska M. Goś R. Jurowski P. (2015). Serum MicroRNAs as potential biomarkers of AMD. Med. Sci. Monit. 21, 2734–2742. 10.12659/MSM.893697 26366973 PMC4576928

[B117] Tai L. Huang C. J. Choo K. B. Cheong S. K. Kamarul T. (2020). Oxidative stress down‐regulates MiR‐20b‐5p, MiR‐106a‐5p and E2F1 expression to suppress the G1/S transition of the cell cycle in multipotent stromal cells. Int. J. Med. Sci. 17, 457–470. 10.7150/ijms.38832 32174776 PMC7053300

[B98] Takada Y. Ogata N. Ohkuma H. Uyama M. (1993). Age-related changes in Bruch’s membrane of the senescence accelerated mouse. Nippon. Ganka Gakkai Zasshi. 97, 595–601. 7687813

[B99] Takada Y. Uyama M. Ohkuma H. Ogata N. Matsushima M. Deguchi J. (1994). Immunohistological study in Bruch’s membrane of senescence accelerated mouse. Nippon. Ganka Gakkai Zasshi. 98, 955–961. 7976831

[B100] Trautner C. Icks A. Haastert B. Plum F. Berger M. (1997). Incidence of blindness in relation to diabetes. A population-based study. Diabetes Care. 20, 1147–1153. 10.2337/diacare.20.7.1147 9203453

[B101] Trip S. A. Schlottmann P. G. Jones S. J. Altmann D. R. Garway-Heath D. F. Thompson A. J. (2005). Retinal nerve fiber layer axonal loss and visual dysfunction in optic neuritis. Ann. Neurol. 58, 383–391. 10.1002/ana.20575 16075460

[B102] Trivino A. Ramírez A. I. Salazar J. J. de Hoz R. Rojas B. Padilla E. (2006). A cholesterol-enriched diet induces ultrastructural changes in retinal and macroglial rabbit cells. Exp. Eye Res. 83, 357–366. 10.1016/j.exer.2005.12.020 16580665

[B103] van den Beld A. W. Kaufman J. M. Zillikens M. C. Lamberts S. W. J. Egan J. M. van der Lely A. J. (2018). The physiology of endocrine systems with ageing. Lancet Diabetes Endocrinol. 6, 647–658. 10.1016/S2213-8587(18)30026-3 30017799 PMC6089223

[B104] van der Schaft T. L. de Bruijn W. C. Mooy C. M. de Jong P. T. (1993). Basal laminar deposit in the aging peripheral human retina. Graefes Arch. Clin. Exp. Ophthalmol. 231, 470–475. 10.1007/BF02044234 8224947

[B105] van der Schaft T. L. de Bruijn W. C. Mooy C. M. Ketelaars D. A. de Jong P. T. (1991). Is basal laminar deposit unique for age-related macular degeneration? Arch. Ophthalmol. 109, 420–425. 10.1001/archopht.1991.01080030122052 2003806

[B106] Wagner H. Fink B. A. Zadnik K. (2008). Sex- and gender-based differences in healthy and diseased eyes. Optometry. 79, 636–652. 10.1016/j.optm.2008.01.024 19811761

[B107] Wagner-Schuman M. Dubis A. M. Nordgren R. N. Lei Y. Odell D. Chiao H. (2011). Race- and sex-related differences in retinal thickness and foveal pit morphology. Invest. Ophthalmol. Vis. Sci. 52, 625–634. 10.1167/iovs.10-5886 20861480 PMC3053303

[B108] Wang C. Ji B. Cheng B. Chen J. Bai B. (2014). Neuroprotection of microRNA in neurological disorders (Review). Biomed. Rep. 2, 611–619. 10.3892/br.2014.297 25053999 PMC4106625

[B109] Wang L. Yang M. Liao S. Liu W. Dai G. Wu G. (2017). Hsa-miR-27b is up-regulated in cytomegalovirus-infected human glioma cells, targets engrailed-2 and inhibits its expression. Exp. Biol. Med. 242, 1535370217699535. 10.1177/1535370217699535 PMC5476335 28343438

[B110] Winkler B. S. Giblin F. J. (1983). Glutathione oxidation in retina: effects on biochemical and electrical activities. Exp. Eye Res. 36, 287–97. 10.1016/0014-4835(83)90013-1 6825742

[B111] Xu W. Li F. Liu Z. Xu Z. Sun B. Cao J. (2017). MicroRNA-27b inhibition promotes Nrf2/ARE pathway activation and alleviates intracerebral hemorrhage-induced brain injury. Oncotarget. 8, 70669–70684. 10.18632/oncotarget.19974 29050310 PMC5642585

[B112] Younan C. Mitchell P. Cumming R. G. Panchapakesan J. Rochtchina E. Hales A. M. (2002). Hormone replacement therapy, reproductive factors, and the incidence of cataract and cataract surgery: the Blue Mountains Eye Study. Am. J. Epidemiol. 155, 997–1006. 10.1093/aje/155.11.997 12034578

[B113] Zarbin M. A. (1998). Age-related macular degeneration: review of pathogenesis. Eur. J. Ophthalmol. 8, 199–206. 10.1177/112067219800800401 9891890

[B114] Zettemberg M. Celojevic D. (2015). Gender and cataract—the role of estrogen. Curr. Eye Res. 40, 176–90. 10.3109/02713683.2014.898774 24987869

[B115] Zetterberg M. (2016). Age-related eye disease and gender. Maturitas. 83, 19–26. 10.1016/j.maturitas.2015.10.005 26508081

