# Corrigendum: Changes in Retinal Structure and Ultrastructure in the Aged Mice Correlate with Differences in the Expression of Selected Retinal miRNAs

**DOI:** 10.3389/fphar.2021.652905

**Published:** 2021-03-15

**Authors:** Anca Hermenean, Maria Consiglia Trotta, Sami Gharbia, Andrei Gelu Hermenean, Victor Eduard Peteu, Cornel Balta, Coralia Cotoraci, Carlo Gesualdo, Settimio Rossi, Mihaela Gherghiceanu, Michele D’Amico

**Affiliations:** ^1^ “Aurel Ardelean” Institute of Life Sciences, Vasile Goldis Western University of Arad, Arad, Romania; ^2^ Department of Biochemistry and Molecular Biology, University of Bucharest, Bucharest, Romania; ^3^ Section of Pharmacology, Department of Experimental Medicine, University of Campania “Luigi Vanvitelli”, Naples, Italy; ^4^ Carol Davila University of Medicine and Pharmacy, Bucharest, Romania; ^5^ Victor Babes National Institute of Pathology, Bucharest, Romania; ^6^ Faculty of Medicine, Vasile Goldis Western University of Arad, Arad, Romania; ^7^ Eye Clinic, Multidisciplinary Department of Medical, Surgical and Dental Sciences, University of Campania “Luigi Vanvitelli”, Naples, Italy

**Keywords:** aging, retina, gender, histology, electron microscopy, miRNAs

In the original article, there was a mistake in Figure 7 as published. The incorrect y-axis header was miR-27a-3p, miR-27b-3p, miR-20a-5p and miR-20b-5p in Figure 7A. The correct y-axis header in Figure 7A is miR-20a-3p, miR-106a-5p, miR-381-3p and miR-206-3p. Moreover, the incorrect miR-20b-5p graph in Figure 7B has been substituted with the correct miR-20b-5p graph. The corrected Figure 7 appears below.

**FIGURE 7:**
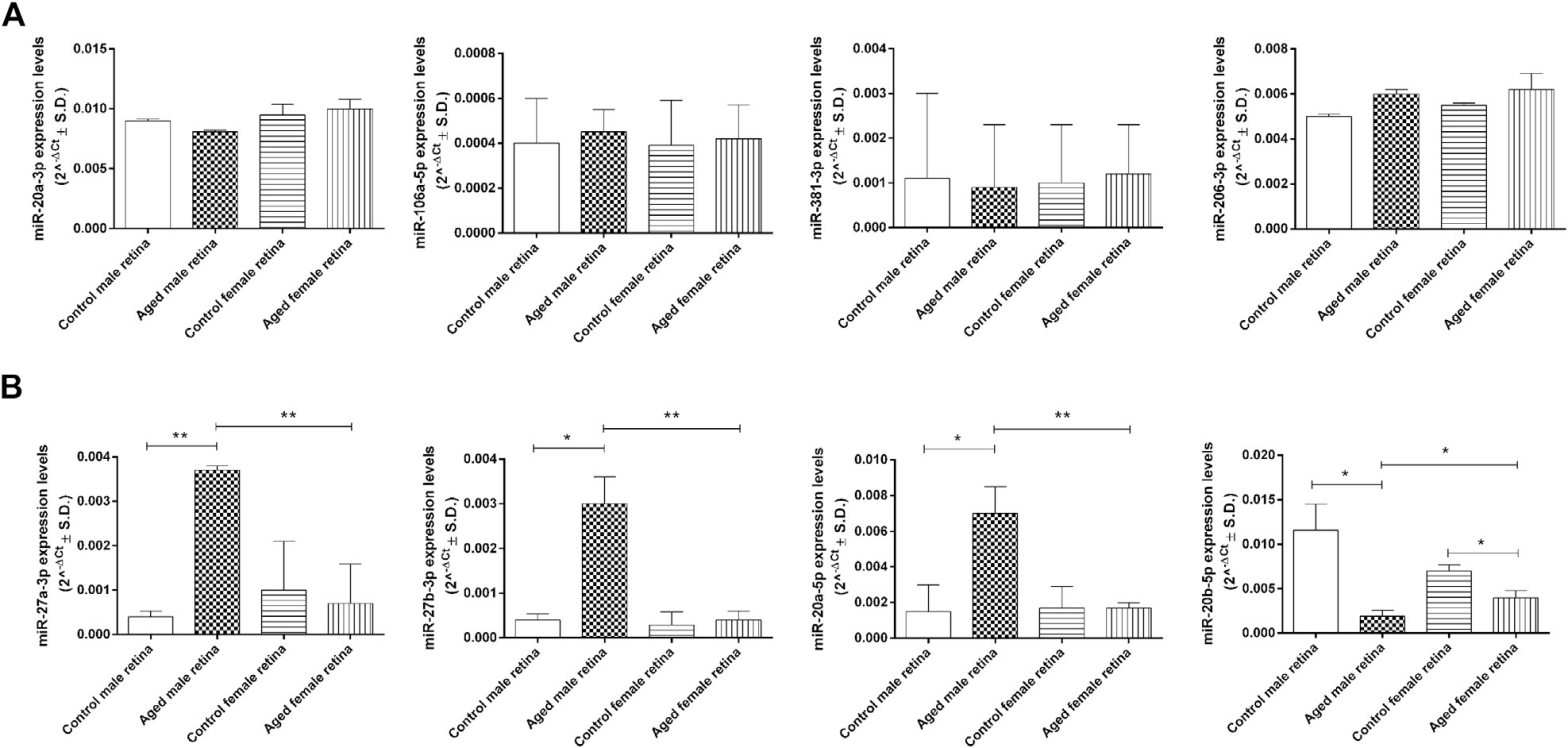
Age-related miRNA expression levels in aged retina. **(A)** Not dysregulated and **(B)** dysregulated retina miRNA expression levels. Data are reported as 2^^−ΔCt^ and shown as mean ± SD of *n* = 10 observations for each experimental group; each run was performed in triplicate. Statistical significance was assessed by using one-way ANOVA, followed by Tukey’s multiple comparisons test. *p < 0.05 and **p < 0.01.

In the original article, there was a mistake in the legend for Figure 9 as published. The incorrect legend caption was “miR-27a-3p, miR-27b-3p, miR-20a-5p, and miR-20b-5p expression levels”. The correct legend caption is “miR-20a-3p, miR-106a-5p, miR-381-3p, and miR-206-3p expression levels”.

In the original article, there was a mistake in Figure 9 as published. The name of each graph was incorrectly reported as miR-27a-3p, miR-27b-3p, miR-20a-5p, and miR-20b-5p. The correct graph names are miR-20a-3p, miR-106a-5p, miR-381-3p, and miR-206-3p. The corrected Figure 9 appears below.

**FIGURE 9:**
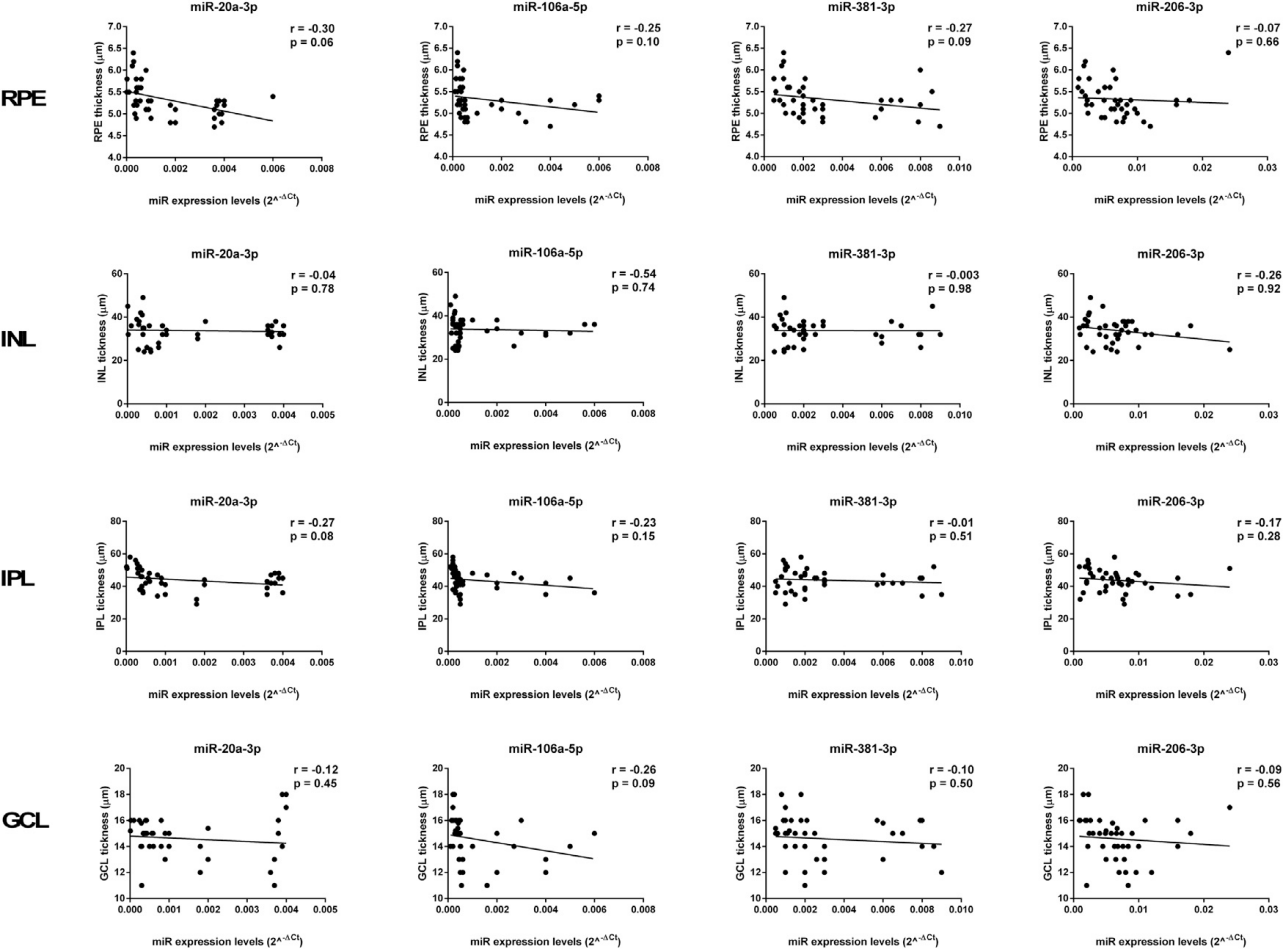
Age-related miRNA expression levels not correlated with retina structure. No significant correlations were observed between RPE, INL, IPL, and GCL thickness and the miR-20a-3p, miR-106a-5p, miR-381-3p, and miR-206-3p expression levels. Pearson correlation analysis was used to evaluate the strength of association between pairs of variables, by including all the samples with different age and gender. Differences were considered statistically significant for *p* values < 0.05. RPE, retinal pigment cells; INL, retinal inner nuclear layer; IPL, retinal inner plexiform layer; GCL, retinal ganglion cell layer.

The authors apologize for these errors and state that this does not change the scientific conclusions of the article in any way. The original article has been updated.

